# The N-terminal domain of the Schaaf–Yang syndrome protein MAGEL2 likely has a role in RNA metabolism

**DOI:** 10.1016/j.jbc.2021.100959

**Published:** 2021-07-12

**Authors:** Matthea R. Sanderson, Richard P. Fahlman, Rachel Wevrick

**Affiliations:** 1 Department of Medical Genetics, University of Alberta, Edmonton, Alberta, Canada; 2 Department of Biochemistry, University of Alberta, Edmonton, Alberta, Canada; 3 Department of Oncology, University of Alberta, Edmonton, Alberta, Canada

**Keywords:** ubiquitination, melanoma antigen, BioID, variant of unknown significance, Prader–Willi syndrome, Schaaf–Yang syndrome, YTHDF2, m^6^A methylation, liquid–liquid phase separation, intrinsically disordered proteins, BioID, proximity-dependent biotin identification, CRAPome, contaminant repository for affinity purification–mass spectrometry data, MAGE, melanoma-associated antigen gene, MHD, MAGE homology domain, LC-MS/MS, Liquid chromatography–tandem mass spectrometry, PWS, Prader–Willi syndrome, SYS, Schaaf–Yang syndrome

## Abstract

*MAGEL2* encodes the L2 member of the melanoma-associated antigen gene (MAGE) protein family, truncating mutations of which can cause Schaaf-Yang syndrome, an autism spectrum disorder. *MAGEL2* is also inactivated in Prader–Willi syndrome, which overlaps clinically and mechanistically with Schaaf–Yang syndrome. Studies to date have only investigated the C-terminal portion of the *MAGEL2* protein, containing the MAGE homology domain that interacts with RING-E3 ubiquitin ligases and deubiquitinases to form protein complexes that modify protein ubiquitination. In contrast, the N-terminal portion of the MAGEL2 protein has never been studied. Here, we find that MAGEL2 has a low-complexity intrinsically disordered N-terminus rich in Pro-X_n_-Gly motifs that is predicted to mediate liquid–liquid phase separation to form biomolecular condensates. We used proximity-dependent biotin identification (BioID) and liquid chromatography–tandem mass spectrometry to identify MAGEL2-proximal proteins, then clustered these proteins into functional networks. We determined that coding mutations analogous to disruptive mutations in other MAGE proteins alter these networks in biologically relevant ways. Proteins identified as proximal to the N-terminal portion of MAGEL2 are primarily involved in mRNA metabolic processes and include three mRNA N 6-methyladenosine (m^6^A)-binding YTHDF proteins and two RNA interference-mediating TNRC6 proteins. We found that YTHDF2 coimmunoprecipitates with MAGEL2, and coexpression of MAGEL2 reduces the nuclear accumulation of YTHDF2 after heat shock. We suggest that the N-terminal region of MAGEL2 may have a role in RNA metabolism and in particular the regulation of mRNAs modified by m^6^A methylation. These results provide mechanistic insight into pathogenic *MAGEL2* mutations associated with Schaaf–Yang syndrome and related disorders.

*MAGEL2* encodes the L2 member of the melanoma-associated antigen gene (MAGE) protein family. Like the 34 other MAGE proteins, MAGEL2 contains a conserved MAGE homology domain (MHD) (1). MAGE proteins regulate protein ubiquitination, through interactions between the MHD and variable domains of E3 ubiquitin ligases and deubiquitinases, to form MAGE-RING E3 ligase complexes that serve as multifunctional hubs for the modification of key substrates in the cell (2, 3, 4). MAGEL2 regulates the vesicular and endosomal trafficking of membrane-bound receptors and also regulates the stability of proteins important for nuclear-cytoplasmic trafficking, cilia, and other cellular activities (5, 6, 7, 8, 9, 10). However, functional studies have assessed only a truncated version of the MAGEL2 protein that contains the C-terminally located MHD but not the N-terminal portion of the protein (6, 7, 8, 11, 12, 13, 14, 15). Despite a growing body of evidence supporting a critical role for MAGEL2 in development and physiology, there is still little known about its cellular role and in particular the role of the N-terminal portion of MAGEL2.

Missense, frameshift, or nonsense mutations in six of the 35 MAGE genes cause genetic disorders: *MAGED2* is mutated in Bartter syndrome (16), *NSMCE3* (*MAGEG1*) in lung disease, immunodeficiency, and chromosome breakage syndrome (17), *MAGEA9* and *MAGEB4* in cases of male infertility (18, 19, 20), while *MAGEA6* mutations promote pancreatic cancer initiation and progression (21, 22). *De novo* or paternally inherited protein-truncating mutations in *MAGEL2* cause MAGEL2-related disorders (Schaaf–Yang syndrome, SYS) (23, 24). Infants with SYS typically present with developmental delay, feeding problems, hypotonia, and joint contractures (25, 26), followed in childhood by intellectual disability, autism spectrum disorder, and endocrine dysfunction (27, 28). Children with SYS have initially been diagnosed with severe hypotonia with respiratory distress (26), recurrent fetal malformations (29), arthrogryposis multiplex congenita, and endocrine dysfunction (30), Chitayat–Hall syndrome (distal arthrogryposis, intellectual disability, dysmorphic features, and hypopituitarism) (31), Crisponi/cold-induced sweating syndrome (hyperthermia, camptodactyly, feeding and respiratory difficulties, scoliosis) (32), hypotonia/obesity syndrome (33), or Opitz trigonocephaly-C (34). A perinatal lethal phenotype is associated with a specific MAGEL2 mutation (c.1996delC, p.Q666Sfs∗36), and moderate to severe phenotypes are associated with the reciprocal mutation, c.1996dupC (p.Q666Pfs∗47, found in 40% of cases) (26, 27, 28, 29, 31, 34, 35, 36, 37, 38, 39). More moderate SYS phenotypes are associated with 39 different protein-truncating mutations located elsewhere in *MAGEL2* (27, 28). As a single exon gene, stop or frameshift mutations in MAGEL2 are not predicted to cause nonsense-mediated RNA decay, and mutant MAGEL2 RNA has been detected at a low level in a human fetus carrying p.Q666Sfs∗36 (37). The *MAGEL2* gene is in the microdeletion region associated with Prader–Willi syndrome (PWS), a neurodevelopmental disorder phenotypically similar to SYS (24).

Missense mutations in MAGE proteins may disrupt protein–protein interactions and function. Mutations in MAGEL2 impair its ability to facilitate retromer-dependent recycling of proteins from endosomes back to the *trans*-Golgi network, to promote the cell surface expression of the leptin receptor and to regulate the ubiquitination and stability of the circadian rhythm protein CRY1 (7, 8, 9). MAGEL2 is most highly expressed in the hypothalamus and to a lesser extent in other regions of the brain and also in the developing musculoskeletal system. Studies in mice also support a critical role for MAGEL2 in the normal development and function of the nervous and endocrine systems, muscle, and bone. Mice carrying paternally inherited *Magel2* mutations have phenotypes reminiscent of SYS, including pre- and perinatal lethality, behavioral abnormalities, abnormal body composition, endocrine dysfunction, low muscle tone, and scoliosis (5, 40, 41, 42, 43, 44, 45, 46, 47, 48, 49, 50, 51, 52).

In this study, we identified proteins in proximity to either the C-terminal portion of MAGEL2 or the entire MAGEL2 protein using *in vivo* proximity-dependent biotin identification (BioID) and affinity capture coupled to liquid chromatography–tandem mass spectrometry (LC-MS/MS) in cultured human cells. We examined how amino acid substitutions in the MHD alter the set of proteins in proximity to MAGEL2, demonstrating the potential utility of protein–protein proximity mapping for the assessment of *MAGEL2* variants of unknown significance in individuals clinically suspected to have SYS. Our study suggests that the N-terminus of MAGEL2 contains an intrinsically disordered domain and associates with proteins that function in mRNA metabolism and cellular stress responses. These results could shed light on the phenotypes associated with both PWS, where there is complete loss of MAGEL2, and SYS, where affected individuals may produce mutant or partial MAGEL2 proteins.

## Results

### Annotation of MAGEL2 functional domains and features

**Figure 1:**
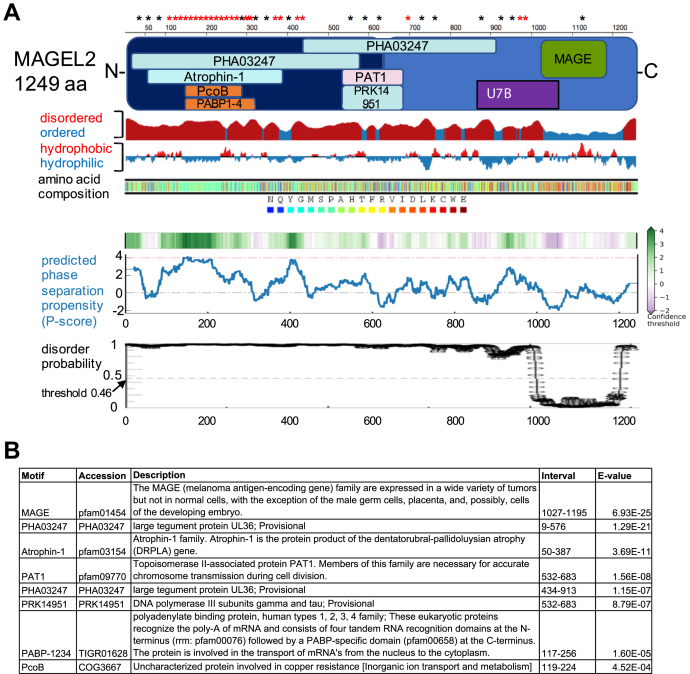
**Analysis of domains and features in the human full-length MAGEL2 protein.***A*, diagram of human MAGEL2 protein (UniProt Q9UJ55) showing location of protein domains and predicted protein structure. Locations of P-G dinucleotides (∗*red*) and P-Xn-G motifs (∗*black*) are indicated at the *top*. Disordered *versus* ordered regions are shown in *red* (potentially disordered region), *blue* (probably ordered region). Hydropathy has been calculated using a sliding window of 15 residues and summing up scores from standard hydrophobicity tables. *Red*: hydrophobic, *Blue*: hydrophilic, adapted from Protein Data Bank: https://www.rcsb.org. Predicted phase separation propensities (PScore) are shown below. A score greater than 4 is considered a confident phase separation prediction and a score of 0 represents the Protein Data Bank average. *B*, domains were predicted by CDD, except for the USP7 binding domain (U7B), which was experimentally determined.

The *MAGEL2* gene was originally predicted to encode a 529 amino acid protein containing a conserved MAGE homology domain (MHD, pfam01454). The DNA upstream of the predicted start codon contains multiple repeated sequences that at the time were not present in cDNA libraries and were refractory to RT-PCR, so this region was assumed to be part of the 5′ untranslated region (11, 53). More recent genome annotation suggests that human MAGEL2 encodes a protein of 1249 amino acid (aa) residues with the MHD from residues 1027 to 1195 (2, 11, 53, 54) (UniProt Q9UJ55) (Fig. 1). A second protein motif, also in the C-terminal portion of MAGEL2, was experimentally determined: U7B, from aa 820 to 1034, binds to the TRAF domain of the ubiquitin-specific protease USP7 (8). We used the NCBI Conserved Domain Database (CDD v.3.17) to analyze MAGEL2 protein. The MHD was a specific hit, representing very high confidence that the query sequence belongs to the same protein family as the sequences used to create the domain model (55). CDD identified seven “non-specific” domains as hits, all of which exceed the default threshold for statistical significance. The portion of MAGEL2 N-terminal to U7B (aa 1–819) is rich in proline (28%), alanine (15%), and glutamine (11%) residues and is predicted to be basic (theoretical isoelectric point 11.5). The N-terminal portion contains two PHA03247 domains, which are low complexity regions rich in alanine, proline, and serine residues (56). CDD also identified a Atrophin-1 domain that contains a polyglutamine repeat (57), a topoisomerase II-associated PAT1 domain (58), a PRK14951 region that is shared with the DNA polymerase III subunits gamma and tau, a PABP-1234 region that is shared among the mRNA-binding PABP proteins, and a PcoB domain, which is present in proteins involved in copper transport. The unusual amino acid composition of the N-terminal region contributes to its predicted instability: its “instability index” is computed to be 79, where a protein whose instability index is ≤40 is predicted as stable (59).

Intrinsically disordered protein regions contain stretches of ≥30 disorder-promoting residues (Arg, Pro, Gln, Gly, Glu, Ser, Ala, and Lys). Using SPOT-Disorder2 (60), we found that about 85% of the MAGEL2 protein, including the entire N-terminal region and a short segment at the extreme C-terminus, exceeds the threshold for an intrinsically disordered protein region (Fig. 1*A*). MAGEL2 is thus one of the 14.5% of human proteins that contain a folded domain (*i.e.*, the MAGE homology domain) and in which intrinsically disordered regions constitute half or more of the protein (61). Intrinsically disordered proteins that are rich in proline and glycine can undergo phase transitions that mediate self-assembly events; examples include elastin-like and collagen-like proteins (61, 62). Protein segments containing proline-glycine pairs separated by up to four residues (P-X_n_-G, where X is any residue), with these motifs separated by 3 to 15 residues, form scaffolds for intrinsically disordered protein polymers. The P-G dipeptide is the predominant motif in these proteins. The N-terminal portion of MAGEL2 is rich in P-X_n_-G motifs, with the portion of MAGEL2 N-terminal to U7B containing 24 P-G dipeptides and 13 other P-X_n_-G motifs (Fig. 1 and Fig. S1*A*). The first 16 P-G dipeptides each start a repeating decapeptide with a consensus sequence approximating P-G-T/V/A-P-M-A/V-H/Q-P-P-P from aa 105 to 263, overlapping the RNA recognition motif (PFAM 00076) of the PABP-1234 domain (63). The average spacing of the P-X_n_-G motifs in the N-terminal portion of MAGEL2 is 15, at the upper end of the range for motifs that form scaffolds for intrinsically disordered protein polymers. Another repeating structure wherein 14 P-P dipeptides each start a repeating heptapeptide with a consensus sequence approximating P-P-P/V/L-I-R-Q-A spans from aa 405 to 502. These repeating proline-rich structures may form scaffolds for intrinsically disordered MAGEL2 polymers (model in Fig. S1*B*). We quantified the propensity of MAGEL2 to contribute to phase separation. MAGEL2 had an overall P-Score of 4.66 using the P-score predictor algorithm, whereby a score ≥4 is considered a confident phase separation prediction, and a score of 0 is average for all proteins in the Protein Data Bank (64). Such low-complexity intrinsically disordered regions can mediate liquid–liquid phase separations to form biomolecular condensates, such as stress granules and P-bodies. As well, proteins associated with autism and neurological disorders are more likely to have higher P-scores than other proteins in the human proteome (61).

### Strategy to identify proteins in proximity to MAGEL2

**Figure 2:**
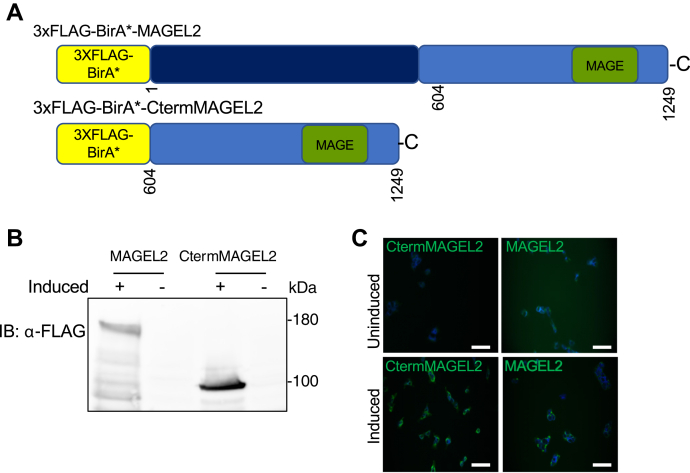
**Generation and expression of BirA∗-MAGEL2 constructs.***A*, a 3XFLAG epitope tag and BirA∗ biotin ligase were fused in frame with the entire 1249 amino acids of MAGEL2 open reading frame or 645 amino acids of the C-terminal end of the MAGEL2 open reading frame. *B*, stably transfected HEK293 cell lines express MAGEL2 when induced with tetracycline. FLAG-BirA∗-MAGEL2 (MAGEL2) and FLAG-BirA∗-CtermMAGEL2 (CtermMAGEL2) proteins were detected in protein lysates from stably transfected HEK293 Flp-In cells induced with tetracycline (+) or not (−) by immunoblotting with anti-FLAG antibodies. *C*, expression of FLAG-BirA∗-MAGEL2 in stably transfected and tetracycline-induced HEK293-MAGEL2 cells plated on coverslips, visualized using anti-FLAG antibodies and confocal microscopy (*green*). Nuclei were counterstained *blue* with Hoechst. Scale bar 50 μm.

We and others previously identified proteins that interact with a C-terminally truncated version of MAGEL2 by yeast two-hybrid screens, affinity capture western, and a mammalian two-hybrid technique called MAPPIT (6, 7, 8, 65, 66). Collectively, these studies identified 12 human and five mouse proteins that interact with the C-terminal portion of MAGEL2 (Table S1). Consistent with functional studies of MAGEL2, the interacting proteins are involved in biological processes such as Arp2/3 complex-mediated actin nucleation, protein K63-linked ubiquitination, and endosome to Golgi retrograde transport. We designed a strategy to identify physiologically relevant proteins in proximity to the entire MAGEL2 protein or to the C-terminal portion, reasoning that we could compare these sets of proteins to implicitly identify proteins in proximity to the unstudied N-terminal portion of MAGEL2. HEK293 Flp-In T-REx cells were stably transfected with a 3xFLAG epitope-tagged-BirA∗ biotin ligase fused in frame to the N-terminus of either the C-terminal 645 residues of the *MAGEL2* open reading frame or to the entire *MAGEL2* open reading frame, creating HEK293-CtermMAGEL2 and HEK293-MAGEL2 cells respectively (Fig. 2*A*). The FLAG-BirA∗ constructs integrate as a single copy into a defined site in the HEK293 Flp-In T-REx genome, and protein expression is inducible with tetracycline in the stable cell line (67, 68). Immunoblotting of lysates from tetracycline-induced cells confirmed the expression of the FLAG-BirA∗ fusion proteins at the expected molecular weight (Fig. 2*B*). The presence of recombinant FLAG-BirA∗-tagged proteins in the cytoplasm of induced cells was detected by indirect immunofluorescence microscopy, consistent with the cytoplasmic localization of endogenous MAGEL2 protein (7, 25) (Fig. 2*C*).

### Functional and Gene Ontology (GO) analyses of CtermMAGEL2 proximal proteins revealed putative MAGEL2 complexes

Expression of FLAG-BirA∗-CtermMAGEL2 was induced in HEK293-CtermMAGEL2 cells cultured in excess biotin. Biotinylated proteins were affinity-purified from cell lysates, processed by tryptic digestion, and analyzed by LC-MS/MS (69). We detected 108 biotinylated proteins that are predicted to have passed within 10 nm of FLAG-BirA∗-CtermMAGEL2 (Table S2). We retained proteins found in at least three of six biological replicate samples and not present at high levels in a contaminant repository for affinity purification–mass spectrometry data (CRAPome, see Experimental procedures) (70). After data processing, 44 CtermMAGEL2-proximal proteins were identified (Table S2). One protein, USP7, had been previously identified as a CtermMAGEL2 interactor by tandem affinity purification and coimmunoprecipitation, while the other proteins were not previously associated with MAGEL2 (8, 9). While we consider these to be potential C-terminal interacting proteins, it is also possible that the N-terminal protein of MAGEL2 could alter the conformation of the C-terminal portion of the protein and influence recruitment of its binding partners.

**Figure 3:**
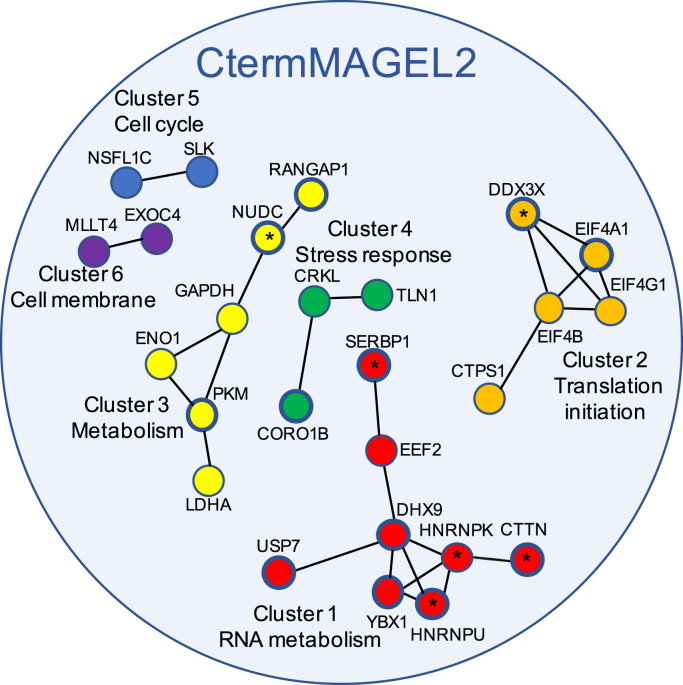
**STRING analysis of proteins in proximity to CtermMAGEL2 as detected by BioID-MS.** The 44 CtermMAGEL2-proximal proteins participate in six clusters of interacting proteins. Proteins later identified as also proximal to the full-length MAGEL2 protein are indicated with a *star*. Proteins with *thicker outlines* were found in all six BioID replicates.

We investigated whether physical interactions among any of the 44 CtermMAGEL2-proximal proteins had been previously detected, using the online tool “STRING: Functional protein association networks” to seed the analysis (71). We did not consider interactions that were based solely on text-mining or coexpression data, focusing on physical interactions, identifying six clusters of CtermMAGEL2-proximal proteins (Fig. 3). Clusters 1 and 2 contain RNA metabolism and translation initiation proteins. Cluster 1 proteins DHX9, YBX1, and HNRNPU associate with IGF2BP1, which binds to the coding region instability determinant of mRNAs and regulates their stability (72). Cluster 2 includes mRNA-binding proteins: eukaryotic initiation factors eIF4A1, eIF4B, and eIF4G are required for the recruitment of the small 40S ribosomal subunit along with other factors that make up the 43S preinitiation complex in preparation for scanning to the AUG codon (73, 74). These eIF proteins interact with DDX3X, a DEAD-box helicase that can substitute for eIF4E in the eukaryotic initiation complex (75). eIF proteins are also components of stress granules (75). Cluster 3 includes the glycolysis proteins GAPDH, ENO1, PKM, and LDHA (76, 77). Cluster 4 is composed of three cellular signaling proteins: CRK-like protein (CRKL), coronin 1B (CORO1B), and talin-1 (TLN1) (78, 79, 80). Cluster 5 proteins regulate the cell cycle: STE20-like kinase (SLK) mediates apoptosis and actin stress fiber dissolution (81), while NSFL1 cofactor p47 (NSFL1C) is important for fragmentation and reassembly of Golgi stacks during cell division (82). Finally, Cluster 6 proteins adafin (MLLT4/ADFN) and exocyst complex component 4 (EXOC4) are PDZ domain-containing proteins that facilitate the formation of cell–cell junctions and docking of vesicles at the cell membrane respectively (83, 84). We next examined whether biological processes or molecular function pathways were enriched among CtermMAGEL2-proximal proteins, using GO algorithms in Cytoscape app (85) (Table S3). As expected, GO classifications mirrored the cores of clusters that were identified by protein–protein interaction analyses in STRING. The category with the most proteins was cadherin binding, followed by ribonucleoprotein complex binding, including subheadings related to translation initiation as well as RNA binding and processing.

### Amino acid substitutions in the MAGE homology domain alter the proximity of proteins to the C-terminal portion of MAGEL2

Mutations in MAGE proteins can disrupt their function, in part because of changes to protein–protein interactions (8, 17). We examined whether CtermMAGEL2 proteins carrying amino acid substitutions in the MHD have different proximal proteins compared with wild-type CtermMAGEL2. Two mutant FLAG-BirA∗-CtermMAGEL2 cDNA constructs were generated by site-directed mutagenesis, then stably transfected into HEK293 Flp-In T-Rex cells to generate cell lines that are isogenic to the wild-type FLAG-BirA∗-CtermMAGEL2 cell line except for the presence of the engineered mutation (Fig. S2*A*). CtermMAGEL2p.R1187C was modeled on a pathogenic missense mutation in a highly conserved arginine residue in the second of two tandem winged helix motifs (WH-B) in the MHD of MAGED2 identified in a patient with Bartter syndrome (4, 16). Mutations in the WH-B of NSMCE3 (MAGEG1) disrupt its ability to bind to double-stranded DNA (86, 87). The double substitution CtermMAGEL2p.LL1031AA (in the WH-A motif) is analogous to engineered mutations in the MAGEG1/NSMCE3 protein that disrupt its ability to bind to the RING-type E3 ubiquitin ligase NSE1 (4, 17). Both MAGEL2 mutations are predicted to alter the structure of the MHD when analyzed using bioinformatic tools (88). These mutations also interfere with MAGEL2 function in ubiquitination assays (7, 9). Induced expression and cytoplasmic localization of each mutant FLAG-BirA∗-CtermMAGEL2 protein were substantiated by immunoblotting of cell lysates (Fig. S2*B*) and immunofluorescence microscopy of tetracycline-induced cells (Fig. S2*C*).

**Figure 4:**
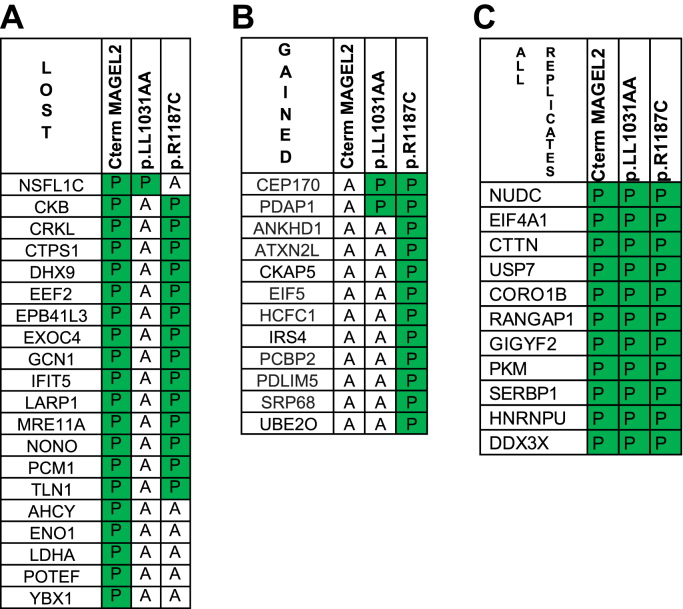
**CtermMAGEL2 proteins carrying amino acid substitutions have losses and gains of proximal proteins compared with wild-type CtermMAGEL2.***A*, proteins present (P) in at least three out of six replicates of BioID-MS with the wild-type CtermMAGEL2 protein but absent (A) in all three replicates of BioID-MS with the mutant CtermMAGEL2 protein are listed. *B*, proteins absent (A) in six out of six replicates of BioID-MS with the wild-type CtermMAGEL2 protein but present (P) in all three replicates with either of the mutant CtermMAGEL2 proteins are listed. *C*, proteins present (P) in all six replicates of the wild-type protein, all three CtermMAGEL2p.LL1031AA replicates, and all three CtermMAGEL2p.R1187C replicates.

Proteins in proximity to CtermMAGEL2p.LL1031AA and CtermMAGEL2p.R1187C were identified by BioID-LC-MS/MS and removal of background proteins, then compared with the set of proteins proximal to wild-type CtermMAGEL2. We defined “lost” interactions as those not detected in any of the three replicates with a mutant CtermMAGEL2 but detected in at least three of six wild-type CtermMAGEL2 replicates, whereas a “gain” of interaction was a proximal protein present in all three replicates for mutant CtermMAGEL2 protein but absent from all six wild-type replicates. Using these definitions, CtermMAGEL2p.LL1031AA had 19 fewer proximal proteins and CtermMAGEL2p.R1187C had six fewer proximal proteins than wild-type CtermMAGEL2 (Fig. 4*A*, Table S2), but CtermMAGEL2p.LL1031AA gained two proximal proteins and CtermMAGEL2p.R1187C gained 12 proximal proteins compared with wild-type CtermMAGEL2 (Fig. 4*B*, Table S2). These results suggest that amino substitutions in the MHD modify the complement of proteins in proximity to MAGEL2. Lastly, nine proteins were proximal to MAGEL2 in all six replicates of the wild-type protein, all three CtermMAGEL2p.LL1031AA replicates, and all three CtermMAGEL2p.R1187C replicates, so represent proximal proteins that are insensitive to these missense mutations in the MAGE homology domain (Fig. 4*C*, Table S2). Six of these proteins are involved in RNA metabolism or translation initiation.

We used STRING to find previously identified interactions among proteins proximal to the two mutant CtermMAGEL2 proteins. Four clusters of proteins were proximal to CtermMAGEL2p.LL1031AA (Fig. S3), while the CtermMAGEL2p.R1187C proximal proteins formed five clusters (Fig. S4). Both mutant proteins maintained interactions with the glycolytic cluster of proteins identified as proximal to wild-type CtermMAGEL2, as well as many of the mRNA binding proteins. However, both MAGEL2 proteins carrying MHD mutations lost interactions with the coding region instability determinant binding proteins YBX1 and DHX9. CtermMAGEL2p.R1187C protein is still proximal to other members of the mRNA-binding core in Cluster 2 containing the proteins eIF4A1, eIF4B, eIF4G1, and DDX3X, while CtermMAGEL2p.LL1031AA maintained proximity to fewer proteins in this cluster. CtermMAGEL2p.R1187C is proximal to more proteins involved in cellular stress response. The CtermMAGEL2p.R1187C Cluster 4 is an expansion of wild-type Cluster 4, with the addition of the adaptor protein crk (CRK). IRS4 is the only member of this cluster not associated with cellular stress response. However, IRS4 is involved in cellular signaling, linking it with the proteins CRK and CRKL (89, 90).

### The full-length MAGEL2 protein interactome is enriched in proteins important for mRNA metabolism

**Figure 5:**
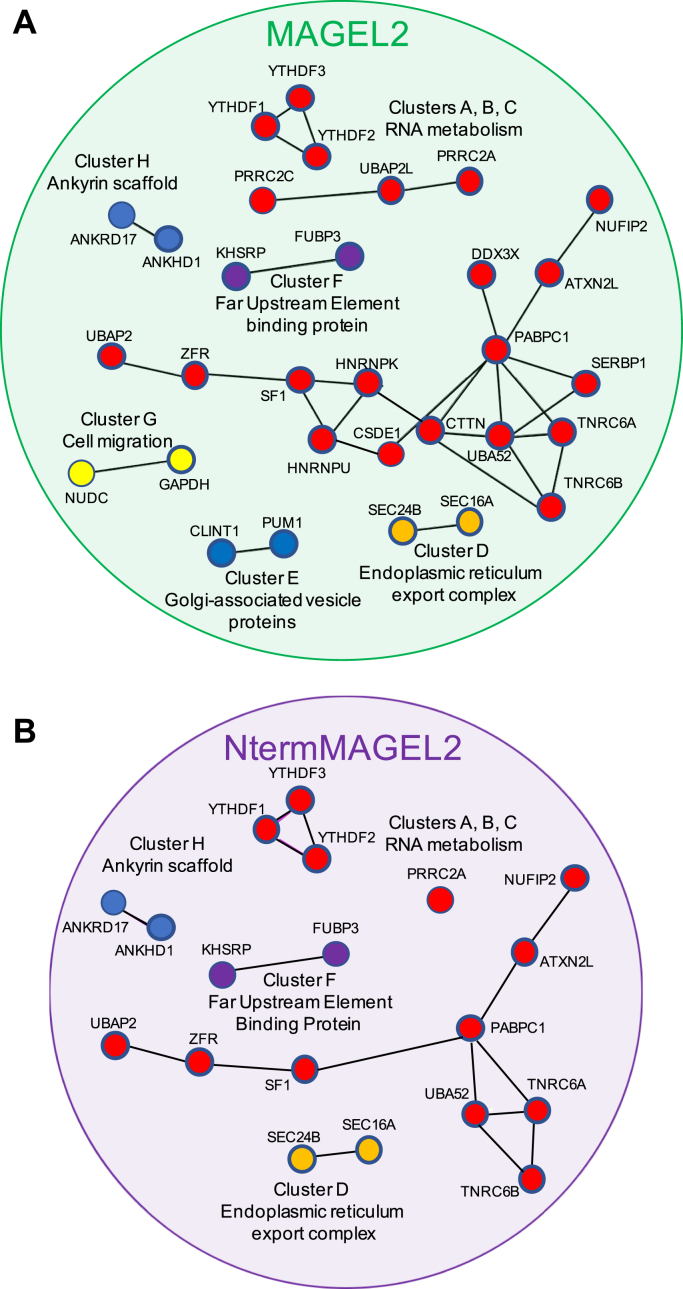
**Protein–protein interaction analysis of proteins in proximity to MAGEL2 as detected by BioID-MS.***A*, the 34 proteins identified in at least two out of three replicate BioID-MS experiments with MAGEL2 and not eliminated as background contaminating proteins were analyzed. Interactions among MAGEL2-proximal proteins were identified, revealing eight clusters of proteins. Proteins outlined with *thicker lines* were found in three out of three replicates. *B*, interactions among proteins proximal to full-length MAGEL2 but not CtermMAGEL2 and deduced to be proximal to the N-terminal, intrinsically disordered portion of MAGEL2. Proteins found in all three replicates of the BioID for full-length MAGEL2 and none of the replicates for C-terminal MAGEL2 are outlined with *thicker lines*.

MAGEL2 encodes a 1249 amino acid protein. However, cellular studies to date have only examined truncated recombinant proteins containing the C-terminal half of MAGEL2 (6, 7, 8, 9, 13, 14, 15). To initiate investigation of the N-terminal portion of MAGEL2, we used BioID to identify proteins in proximity to the full-length MAGEL2 protein. Comparing these full-length MAGEL2 proximal proteins to proteins proximal to the C-terminal portion implicitly identifies proteins proximal to the unstudied N-terminal portion of MAGEL2. Expression of FLAG-BirA∗-MAGEL2 (*i.e.*, full length) was induced in HEK293-MAGEL2 cells cultured in excess biotin, and 34 proximal proteins were identified by BioID-LC-MS/MS and elimination of contaminants (Table S2). These MAGEL2-proximal proteins form eight clusters of proteins by STRING analysis (Clusters A–H, Fig. 5*A*). Cluster A contains a set of RNA binding and processing proteins, including polyadenylate-binding protein 1 (PABPC1), a protein that binds to the poly (A) tail of mRNA and regulates it through splicing and stability (91, 92). Other proteins in Cluster A are also involved in mRNA metabolism: CSDE1, DDX3X, HNRNPK, HNRNPU, TNRC6A, TNRC6B, SERBP1, and ZFR (93, 94, 95, 96, 97, 98). Cluster B is made up of Ubiquitin-associated protein 2-like (UBAP2L), proline-rich coiled-coil 2 A (PRRC2A) and PRRC2C, which bind and process RNA and are core stress granule proteins (99, 100) PRRC2A is also a m^6^A reader protein (101). N^6^-methyladenosine (m^6^A) is the most common eukaryotic mRNA modification, and m^6^A RNA modification is recognized and bound by reader proteins that regulate mRNA stability and the rate of mRNA translational output (102, 103). Regulation of m^6^A dependent processes is important for neurogenesis and synaptic function, and dysregulation occurs in disorders such as Fragile X syndrome. Cluster C has YTHDF1, YTHDF2, and YTHDF3 (YT521-B homology Containing Family 1/2/3), which are important for the translation and degradation of m^6^A modified mRNAs (102, 104, 105). In Cluster D, SEC16A and SEC24B function in export of cargo from the endoplasmic reticulum (106, 107, 108). PUM1 (Pumilio homolog 1) and CLINT1 (Clathrin interactor 1) are Golgi-associated vesicle interacting proteins that form Cluster E (109). PUM1 binds to mRNA and directs it to be repressed or translated (110), and CLINT1 plays a role in clathrin-coated vesicles from the *trans*-Golgi network (111). FUBP3 and KHSRP (FUBP2) are far upstream element (FUSE) binding proteins that bind RNA and single-stranded DNA and form Cluster F. In Cluster G, the chaperone protein (NUDC) plays a role in neuronal cell migration (112), while glyceraldehyde-3-phosphate dehydrogenase (GAPDH) is a metabolic protein. Cluster H contains ankyrin scaffold proteins ANKHD1 and ANKRD17, which regulate nucleocytoplasmic trafficking of the transcriptional regulator YAP and cytokine receptor signaling.

### The full-length MAGEL2 interactome contains proteins that are not in the C-terminal MAGEL2 interactome and are inferred to be proximal to the N-terminal portion of MAGEL2

Ideally, we would next identify proteins in proximity to the N-terminal region of the MAGEL2 protein, expressed without the C-terminal region. However, this experiment is complicated by the unusual amino acid composition of the N-terminal region that renders it prone to aggregation, unstable, and likely to mislocalize in the cell. Instead, we compared the 34 proteins proximal to full-length MAGEL2 to the 44 proteins proximal to the C-terminal portion of MAGEL2, identifying 12 proteins common to both datasets and inferred to be proximal to C-terminal MAGEL2. There were 22 proteins new to the full-length MAGEL2 interactome and not found in any of the six replicate BioID experiments with C-terminal MAGEL2, suggesting that these proteins are in proximity to the N-terminal portion of MAGEL2 (Fig. 5*B*, Fig. S5, Table S4). Alternatively, some of these proteins could be proximal to the C-terminal region of MAGEL2 only when the N-terminal region is present, as a result of a change in conformation or subcellular localization. Functional pathways enriched among presumed N-termMAGEL2 proximal proteins were identified using associated GO terms (85) (Table S5). Nineteen of the 22 proteins are involved in transcription or mRNA processing, including many that interact with each other (Fig. 5). TNRC6A and TNRC6B and YTHDF1, YTHDF2, and YTHDF3 are RNA-binding proteins (104, 113). PABPC1 (polyadenylate-binding protein C1) is one of several PABP proteins that have roles in RNA metabolic pathways, including recognition of poly(A) tails of mRNAs and mRNA transport from the nucleus to the cytoplasm (114). PABPC1 also binds TNRC6A (115). Interestingly, the N-terminal portion of MAGEL2 itself contains a PABP domain, between residues 117 and 256, suggesting that the MAGEL2 N-terminal domain may function as a PABP-like protein.

YTHDF2 was proximal to MAGEL2 in all three replicate BioID experiments. A previous comprehensive study of mRNA-associated processes or complexes by BioID proximity mapping identified 100 proteins as high-confidence interactors of YTHDF2 (100, 104). Of the 22 proteins deduced to be in proximity to the N-terminal portion of MAGEL2, 14 were among the 100 high-confidence YTHDF2 interactors (Fig. S6). As well, 13 of these proteins are among the top 25 interactors for the set of YTHDF1, YTHDF2, and YTHDF3 proteins (104). MAGEL2 is not endogenously expressed in HEK293 cells used for BioID, so was not identified as a YTHDF interactor. However, the MAGE family member MAGED1 was one of the top 100 YTHDF2 interacting proteins (Fig. S6). This observation, and the fact that mRNA-binding proteins are also enriched in the interactome of necdin, a highly related MAGE protein (88), raises the possibility that MAGE proteins MAGEL2, necdin, and MAGED1 might serve redundant roles in mRNA-associated complexes.

### MAGEL2 complexes with YTHDF proteins, and YTHDF2 levels are reduced in PWS fibroblasts

**Figure 6:**
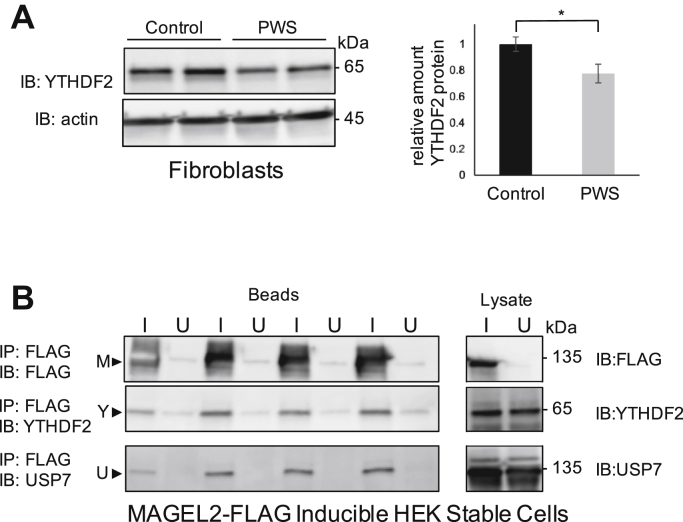
**Human fibroblasts from individuals with PWS have less YTHDF2 protein than control fibroblasts, and endogenous USP7 and YTHDF2 proteins coimmunoprecipitate with MAGEL2.***A*, endogenous YTHDF2 protein levels were measured by immunoblotting lysates from cultured immortalized fibroblasts. C12 and C14 are fibroblast lines from control individuals, whereas P16 and P21 are fibroblast cell lines from individuals with PWS lacking MAGEL2 expression. *Right*, analysis of replicate blots of YTHDF2 levels in control and PWS fibroblasts, with equal amounts of total protein loaded per lane (mean ± standard error of the mean, n = 8 per genotype, ∗*p* = 0.03 by Student *t* test). *B*, quadruplicate samples of MAGEL2-HEK cells were induced (I) or uninduced (U) to express full-length MAGEL2 (M). Endogenous YTHDF2 and endogenous USP7 coimmunoprecipitated with FLAG-MAGEL2 using anti-FLAG M2 gel. 10% of each cell lysate was immunoblotted to confirm the presence of all proteins in the input lysates.

The C-terminal portion of MAGEL2 is an adaptor for protein ubiquitination, a posttranslational modification that alters protein stability (6, 7, 9). YTHDF2 undergoes ubiquitin-mediated proteolysis in a cell-cycle-dependent manner (116). In a cotransfection experiment, we found that coexpression of MAGEL2 increased the steady-state levels of YTHDF2 (Fig. S7). Moreover, we found less endogenous YTHDF2 protein in fibroblasts derived from individuals with PWS, who lack MAGEL2, *versus* control fibroblasts (Fig. 6*A*). Next, given that BirA∗-MAGEL2 proximity labels YTHDF1, YTHDF2, and YTHDF3, MAGEL2 and YTHDF proteins may be near enough to each other to form protein complexes. Indeed, endogenous YTHDF2 coimmunoprecipitated with FLAG-MAGEL2 produced in tetracycline-induced stable HEK293-MAGEL2 cells (Fig. 6*B*). As a control, endogenous USP7 also coimmunoprecipitated with MAGEL2 (Fig. 6*B*) (8). Transient transfection and immunoprecipitation of FLAG-tagged YTHDF1, YTHDF2, or YTHDF3 coimmunoprecipitated coexpressed V5-MAGEL2, demonstrating that MAGEL2 and each of the three YTHDF proteins can form protein complexes, at least in this heterologous expression system (Fig. S8). However, recombinant V5-tagged CtermMAGEL2 did not coimmunoprecipitate with any of the YTHDF proteins, consistent with BioID results that showed YTHDF proteins in proximity to full-length MAGEL2 but not the C-terminal portion of MAGEL2. We also expected that MAGEL2 and YTHDF proteins should be present in the same cellular compartments. We transiently cotransfected U2OS cells, which have a larger cytoplasmic compartment than HEK293 cells so allow for improved visualization of recombinant proteins by confocal microscopy. MAGEL2 and each YTHDF protein are present diffusely in the cytoplasm, with signals overlapping in the cytoplasmic and perinuclear region of the cell (Fig. S9).

### MAGEL2 has an expanded number of interactions after heat shock stress

YTHDF proteins are found in the cytosol and the nucleus, and all three YTHDF proteins partially relocalize to stress granules under stress conditions (117). To determine whether MAGEL2 protein–protein interactions are sensitive to stress, we induced MAGEL2 expression in HEK293-MAGEL2 cells, treated with biotin, heat shocked the cells at 42 °C for 1 h, then processed the cell lysates for BioID-MS. After eliminating proteins found in only one of three replicate samples or present at high levels the CRAPome, 364 MAGEL2-proximal proteins were identified in heat-shocked HEK293-MAGEL2 cells, including 33 of the 34 MAGEL2 proximal proteins identified under nonstressed conditions (Table S2). The heat-shocked MAGEL2 proximity interactome was enriched for ribosomal proteins and RNA-binding processes such as mRNA metabolism and mRNA splicing. In a parallel experiment, 768 proximal proteins enriched for ribosomal proteins and proteins involved in mRNA metabolism and translation initiation were in proximity to CtermMAGEL2 after heat shock (Table S2).

**Figure 7:**
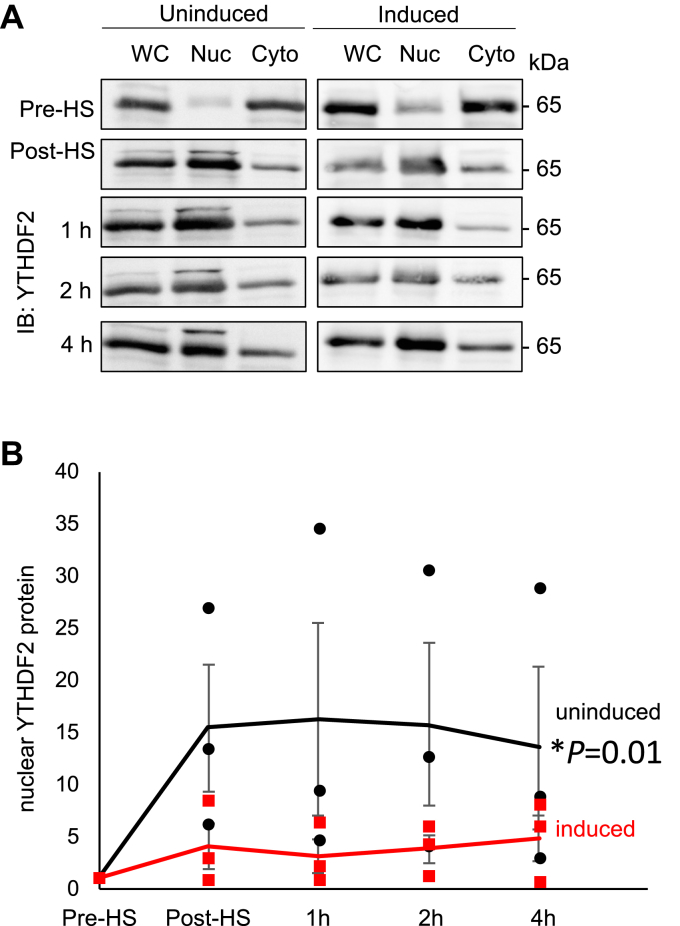
**YTHDF2 protein levels in the nucleus change in response to heat shock, and MAGEL2 expression blunts this response.***A*, HEK293-MAGEL2 cells that were either induced with tetracycline to express MAGEL2, or uninduced, were placed in a 42 °C water bath for 1 h. Cells were harvested at different time points, before heat shock (Pre-HS), immediately following heat shock (Post-HS) or at 1, 2, and 4 h following removal of the samples from the water bath. Proteins from cellular fractions (whole cell, WC, or nuclear fraction, Nuc, or cytoplasmic fraction, Cyto) were immunoblotted to examine the abundance of endogenous YTHDF2. Equal amounts of protein were loaded. A representative trial is shown. *B*, the change in the proportion of YTHDF2 in the nucleus relative to preheat shock in induced (MAGEL2-expressing) and uninduced cells was plotted over time for triplicate samples, normalized to the amount of YTHDF2 in the nuclear fraction a pre-heat shock (uninduced, *black* and induced, *red*, mean ± standard deviation, individual triplicate values are also shown). The relative amount of YTHDF2 protein in the nucleus increases after heat shock in uninduced (*black*, no MAGEL2 expression) HEK293-MAGEL2 cells (repeated measures ANOVA, ∗*p* < 0.01, n = 3). There was no change in relative levels of nuclear endogenous YTHDF2 levels after heat shock in induced, MAGEL2-expressing cells (*red circles*, repeated measures ANOVA, ∗*p* > 0.05, n = 3).

We next asked whether coexpression of MAGEL2 might modify the responses of YTHDF2 to heat shock. YTHDF2, an m^6^A “reader,” regulates 5′UTR methylation and translation initiation under conditions of stress, in part by relocalizing to the nucleus to prevent demethylation by m^6^A “erasers” (118). Expression of MAGEL2 was induced (or not) in HEK293-MAGEL2 cells, then cells were heat shocked in a 42 °C water bath for 1 h and harvested at time intervals thereafter. Cells were fractionated into nuclear and cytoplasmic fractions, in three replicate trials (119) (Fig. 7*A*). The amount of nuclear YTHDF2 protein under each condition was normalized to the whole cell protein and plotted over time (Fig. 7*B*). In response to heat shock, the proportion of YTHDF2 found in the nuclear fraction after heat shock increased in uninduced HEK293-MAGEL2 cells, compared with before heat shock (repeated measures ANOVA, F = 7.7 F crit = 4.4, n = 3, *p* = 0.01). However, induction of MAGEL2 expression in HEK293-MAGEL2 prevented the redistribution of YTHDF2 protein into the nucleus after heat shock (*p* > 0.05).

## Discussion

*MAGEL2* is mutated in people with SYS and inactivated in people with PWS, but how the loss of MAGEL2 function contributes to pathophysiology these disorders is unknown. Identifying the proteins proximal to MAGEL2 could shed light into the function of this enigmatic protein, and particularly the function of the N-terminal region of MAGEL2 that has not previously been studied. Our bioinformatic analysis revealed that the N-terminal portion of MAGEL2 contains intrinsically disordered protein domains with a highly predicted propensity toward protein phase separation. We used proximity-based biotinylation (BioID) coupled with mass spectrometry to identify MAGEL2-proximal proteins, many of which were previously shown to physically associate with each other. The C-terminal proximal proteins formed clusters of interacting proteins that function in RNA metabolic processes, stress response, and metabolism. Proximal proteins were enriched for GO terms such as cadherin binding, ribonucleoprotein complex binding, translation initiation, and RNA binding and processing. When merged with processes ascribed to previously identified MAGEL2-interacting proteins, such as Arp2/3 complex-mediated actin nucleation, ubiquitination, and retrograde transport, our study points to possible novel roles for CtermMAGEL2.

The proteins proximal to CtermMAGEL2 differed when structure-altering mutations were made in the C-terminal MAGE homology domain. For example, the coding region instability determinant binding proteins YBX1 and DHX9 were proximal to wild-type MAGEL2, but were not proximal to the MHD-mutated MAGEL2 proteins. We recently demonstrated that analogous mutations in the MHD of necdin, another MAGE protein, alter the necdin protein interactome (88). Interestingly, MAGE proteins carrying an arginine to cysteine MHD mutation, that is, MAGEL2p.R1187C and NDNp.R265C modeled on the Bartter syndrome mutation MAGED2p.R446C, had more BioID-identified proximal proteins than wild-type ((88) and this report). In contrast, both CtermMAGEL2p.LL1031AA (this study) and NDNp.VL109AA (88) had fewer proximal proteins than their wild-type counterparts. These changes in interaction could be physiologically relevant, given that pathogenic mutations in the MHD of MAGEG1/NSMCE3 cause a chromosome breakage syndrome by disrupting interactions with NSCME4, thus destabilizing the SMC5/6 complex (17, 120). BioID-MS could be a valuable tool for the evaluation of coding variants of unknown significance in *MAGEL2* or other MAGE genes associated with disease (16, 17, 18, 19, 20, 21, 22, 23, 24).

The most novel part of our study is the analysis of proteins proximal to the N-terminal region of MAGEL2. Proteins with high phase separation prediction values are preferentially associated with GO terms such as phase-separated compartments (*e.g.*, stress granules and postsynaptic densities), in RNA processing, in the assembly and plasticity of structural components, and in signaling, regulation, and development (64). MAGEL2-proximal proteins are highly associated with an overlapping set of GO terms, in particular RNA processing activities, consistent with the high phase separation prediction score that we identified for the N-terminal region of MAGEL2. Two sets of RNA-binding proteins, TNRC6A and TNRC6B (trinucleotide repeat containing adaptor 6 A/B), and YTHDF1, YTHDF2, and YTHDF3 (YTH N^6^-methyladenosine RNA-binding protein 1/2/3), were proximal to full-length MAGEL2 but not to C-terminal MAGEL2. TNRC6A and TNRC6B are paralogs of the *Drosophila* GW182 scaffolding proteins and act in posttranscriptional gene silencing through RNAi and microRNA pathways. TNRC6A/B complexes with mRNAs and Argonaute proteins in P-bodies in the cytoplasm and can recruit CCR4-NOT and PAN deadenylase complexes to repress mRNAs (113).

Complexes containing YTHDF proteins and m^6^A-modified mRNA partition into cellular phase-separated compartments that differentially regulate the stability and translation of the mRNAs (104, 117). YTHDF proteins have a conserved interaction domain in their C-terminal portion, namely the YTH domain that binds polymethylated m^6^A modified mRNAs. They also have a low-complexity prion-like domain that contains P-X_n_-G motifs, in their N-terminus (103, 117, 121). Like other low-complexity proteins, YTHDF proteins interact with each other and undergo warming-induced liquid–liquid phase separation, which allows them to form membraneless biomolecular condensates such as stress granules and processing bodies (P-bodies) (62, 122, 123). YTHDF2 regulates 5′UTR methylation and translation initiation under conditions of stress, in part by relocalizing to the nucleus to prevent demethylation by m^6^A “erasers” (84, 118, 121). We show that MAGEL2 interacts with YTHDF2 by BioID and coimmunoprecipitation. In addition, we found that in response to heat stress, YTHDF2 moves to the nucleus. However, MAGEL2 expression abrogated the increase of YTHDF2 in the nucleus after heat shock. These data suggest that MAGEL2 could regulate the translocation of YTHDF2 to the nucleus, by an as yet unknown mechanism (124).

Like YTHDF proteins, MAGEL2 contains Pro-X_n_-Gly motifs in its N-terminal region, which is predicted to be an intrinsically disordered domain. MAGEL2 is predicted to undergo phase separation and localize to membraneless compartments in the cell, perhaps accounting for the large number of proteins in proximity to MAGEL2 under conditions of stress. Interestingly, the N-terminal portion of MAGEL2 has more P-X_n_-G motifs and a smaller spacing between them than the YTHDF proteins. In contrast elastin, a prototypical P-X_n_-G intrinsically disordered self-assembling protein, has a denser arrangement of P-X_n_-G motifs than the N-terminal domains of either MAGEL2 or YTHDF. The most common mutations in SYS cause a frameshift at glutamine at position 666, potentially resulting in the production of a truncated protein encoding only the Pro-X_n_-Gly-rich, intrinsically disordered N-terminus of MAGEL2. A MAGEL2 truncated protein could impair the dynamics of ribonucleoprotein bodies that undergo liquid–liquid phase separation. This supports a model that SYS-causing frameshift mutations encode a truncated MAGEL2 protein that could be prone to pathological aggregation in RNA-rich granules when no longer associated with the structured C-terminus. There is no biological evidence that this occurs in individuals with SYS, in part because MAGEL2 is only expressed in discrete regions of the brain. However, we did previously find changes in the abundance of p62-rich cytoplasmic inclusions in muscle and hypothalamus of mice carrying a mutation in the *Magel2* gene that results in a truncated Magel2 protein (5). A toxic aggregation model would also explain the more severe and sometimes fatal outcomes associated with MAGEL2 frameshift and stop mutations causing SYS, compared with the typical presentation in PWS, in which the MAGEL2 gene is inactivated completely and MAGEL2 protein is absent (24).

Notably, the missense mutations that we investigated were in the MAGE homology domain in the folded region of MAGEL2. Mutations in intrinsically disordered regions of proteins, such as the N-terminal region of MAGEL2, are more challenging to evaluate (61). Many autism spectrum disorder susceptibility genes encode proteins that function in RNA processing, activity-dependent translation, and synaptic function (125). Furthermore, both phase separation and the partitioning of proteins in the cell can be disrupted by pathogenic mutations in these susceptibility genes (61). Given that frameshift mutations in MAGEL2 cause autism spectrum disorder, our study raises the possibility that an untapped reservoir of mutations in the intrinsically disordered N-terminus of MAGEL2 could contribute to some of the missing heritability in neurodevelopmental disorders.

Our study has some limitations. BioID can detect weak, transient, or indirect protein–protein interactions, so the proteins identified as part of the MAGEL2 interactome may reside at a further distance from MAGEL2 than proteins identified as direct interactors by other methods. The HEK293-Flp-In cell line used for these experiments was ideal for comparison of BioID results for different MAGEL2 proteins because of the single integration site and ability to induce low level expression of MAGEL2, in contrast to overexpression systems. While HEK293 cells have some neuronal phenotypes (126), they do not normally express *MAGEL2*, so some proximal proteins may not be physiologically relevant in the tissues where MAGEL2 is normally expressed, such as the brain, muscle, and bone. The use of recombinant proteins and the location of the BirA∗ tag were also limitations of the study. MAGEL2 proteins carried N-terminal BirA∗ tags, which was ideal for comparison among the variant MAGEL2 proteins, but complementary studies using C-terminally tagged MAGEL2 proteins would be informative. To further expand our knowledge of the MAGEL2 interactome, we compared proteins proximal to the full-length MAGEL2 protein, which has not been studied previously, to proteins proximal to the C-terminal half of MAGEL2. The results suggest that the N- and C-terminal domains of MAGEL2 are both important for protein interactions. It is unclear why there are certain proteins proximal to CtermMAGEL2 and not the full-length protein. It is possible that without the N-terminal portion of the protein, the MHD is more available to bind proteins. Alternatively, the N-terminus could obscure proteins that complex with the C-terminus, or the unstructured nature of the N-terminus could increase the volume that the protein occupies, putting the C-terminus out of the 10 nm labeling range of the biotin ligase (127). Unfortunately, we were not able to express the N-terminal region of the protein alone, nor test whether presumptive N-terminal proximal proteins indeed interact with the N-terminus of the protein.

In conclusion, we used proximity-dependent labeling and mass spectrometry to identify novel proteins in proximity to MAGEL2. When we analyzed the proximal MAGEL2 and CtermMAGEL2 together using STRING, we found that they form a large network of proteins that function in translation initiation and ubiquitination. The 13 proteins that are proximal to both MAGEL2 and CtermMAGEL2 represent the proteins that are the highest confidence for being direct interactors of MAGEL2. The 22 proteins that interact with only the full-length protein represent novel subjects for the focus of MAGEL2 functional experiments, particularly in the area of RNA processing and phase separation. Further studies are needed to determine whether BioID-MS can be used to examine the functional impact of MAGEL2 missense mutations identified in individuals carrying a clinical diagnosis of SYS. The functional pathways identified among MAGEL2-proximal proteins indicate potential avenues of investigation to understand the cellular functions disrupted in both PWS and SYS.

## Experimental procedures

### Bioinformatic protein analysis

The NCBI Conserved Domain Database (CDD v.3.17) was used to analyze MAGEL2 protein (CCDS73700) for motifs (55). Protein Data Bank (128) Protein Feature View (https://www.rcsb.org) was used to visualize disordered *versus* ordered regions (computed by JRONN (129)) and hydropathy, calculated using a sliding window of 15 residues and summing up scores from standard hydrophobicity tables. Amino acid composition was visualized using PLAAC (Prion-like amino acid composition) (130). Predicted phase separation propensities (PScore) were calculated using Pscore Predictor (64). Probability scores for intrinsically disordered protein regions were calculated using SPOT-Disorder2, with a score >0.46 indicating an intrinsically disordered protein region (60). Identification of Pro-X_n_-Gly motifs in proteins was performed in MATLB using Script 1 (62).

### Plasmid construction

pENTR clones containing CtermMAGEL2p.LL1031AA and CtermMAGELp.R1187C were created in the wild-type pENTR-CtermMAGEL2cDNA (DNASU Clone: HsCD00295122, NCBI Reference Sequences: NM_019066.5 and NP_061939.3) by site-directed mutagenesis. Mutations in MAGEL2 were as follows: p.Leu1031_Leu1032delinsAlaAla (p.VL1031AA, c.[C1281CG;T1282C;C1284G;T1285C]), and p.Arg1187Cys (p.R1187C, c.[C1750T;A1752T]. In brief, tail-to-tail oligonucleotide primers containing the desired missense mutation were used for PCR amplification of pENTR-CtermMAGEL2. The PCR product was digested with DpnI, phosphorylated, and recircularized by ligation. The presence of the respective mutations was confirmed by sequencing. MAGEL2, CtermMAGEL2, and mutant CtermMAGEL2 cDNAs were transferred to pDEST-pcDNA5-BirA∗-FLAG using gateway recombinational cloning to create FLAG-BirA∗-MAGEL2, FLAG-BirA∗-CtermMAGEL2, and mutant FLAG-BirA∗-CtermMAGEL2 constructs (7, 67). FLAG-YTHDF1, FLAG-YTHDF2, and FLAG-YTHDF3 were provided by Dr Chuan He (131). Plasmids pOG44 and pDEST-pcDNA5-BirA∗-FLAG were provided by Dr A-C Gingras (67).

### Cell culture, cell lines, and transfections

Tissue culture reagents were from Thermo-Fisher Scientific unless otherwise stated. Flp-In T-Rex HEK293 cells (Invitrogen) were cultured in Dulbecco’s modified Eagle medium (DMEM) supplemented with 10% fetal bovine serum, 1% L-glutamine, and 1% penicillin/streptomycin at 37 °C with 5% CO_2_, and maintained in zeocin (100 μg/ml) and blasticidin (15 μg/ml) (132, 133). To generate stable cell lines, Flp-In T-Rex HEK293 cells were seeded at a density of 1 × 10^5^ cells per well in a 6-well plate and cotransfected 24 h later with pOG44 and pcDNA FLAG-BirA∗ constructs at a ratio of 9:1 using FuGENE6 (Promega E2691). Cells were then cultured in media without zeocin or blasticidin, but with hygromycin B (100 μg/ml) to select for cells carrying stable integration of the construct. These cell lines, carrying FLAG-BirA∗-MAGEL2 constructs, are called HEK293-MAGEL2, HEK293-CtermMAGEL2, or HEK293-mutantCtermMAGEL2. For heat shock experiments, HEK293-MAGEL2 cells that had or had not been induced for MAGEL2 expression were placed in a 42 °C water bath for 1 h. Lysates were harvested at different time points, before heat shock, immediately following heat shock, and then at 1 h, 2 h, and 4 h following removal from the water bath. Cellular fractionation was performed on the lysates using the REAP 2 min nonionic detergent-based purification technique (119). Human control fibroblasts (FB12 NIGMS repository number GM01601A, FB14 GM00650) were from the NIGMS human genetic cell repository (Camden NJ). PWS fibroblasts (15q11-q13 deletion) were FB16 (1889 PWS) from the Brain and Tissue Banks for Developmental Disorders, and PWS129 from Dr Daniel Driscoll University of Florida Gainesville, and RCB1560 from RIKEN Bioresource Centre Cell Bank in Tsukuba Japan. Cell lines are regularly tested for *mycoplasma*.

### Proximity-dependent biotin identification coupled to affinity capture and mass spectrometry

Stably transfected HEK293-MAGEL2, HEK293-CtermMAGEL2, or HEK293-mutantCtermMAGEL2 cells were cultured in 10 cm dishes and incubated overnight with 1 μg/ml tetracycline and 50 μM biotin. Cells were washed twice with 10 ml PBS to remove excess biotin prior to collection. BioID analysis was adapted from previously described methods (7, 68), with the following modifications: cells were lysed in 2.2 ml of lysis buffer (50 mM Tris HCl, 500 mM NaCl, 0.2% SDS, 2% Triton-X, pH 8.0, 1× Complete Mini Protease Inhibitor (Roche)), 150 μl of streptavidin sepharose beads were used in the affinity capture, and the beads were washed four times with 100 mM ammonium bicarbonate (AmBic), then reduced (10 mM beta-mercaptoethanol in 100 mM AmBic) and alkylated (55 mM iodoacetamide in 100 mM AmBic). Trypsin (150 μl of 6 ng/μl, Promega Sequencing grade, specifically cleaves at the carboxylic side of lysine and arginine residues) was added, and the digestion was allowed to proceed overnight (∼16 h) at 30 °C. The supernatant was collected and the beads were washed with 100 μl of extraction buffer (97% water/2% acetonitrile/1% formic acid) followed by a second 100 μl wash with 50% extraction buffer and 50% acetonitrile. Both washes were combined with the initial supernatant, and the samples were then dried under vacuum and then dissolved in 80 μl 0.3% formic acid.

Samples were resolved and ionized by using nanoflow HPLC (Easy-nLC II, Thermo Scientific) with a PicoFrit fused silica capillary column (ProteoPepII, C18) with 100 μm inner diameter (300 Å, 5 μm, New Objective) coupled to an LTQ XL-Orbitrap hybrid mass spectrometer (Thermo Scientific). Peptide mixtures were injected (10 μl) onto the column and resolved at 500 nl/min using a 60 min linear gradient from 0 to 35% v/v aqueous ACN in 0.2% v/v formic acid. The mass spectrometer was operated in data-dependent acquisition mode, recording high-accuracy and high-resolution survey Orbitrap spectra using external mass calibration, with a resolution of 30,000 and m/z range of 400 to 2000. The 14 most intense multiply charged ions were sequentially fragmented by using collision induced dissociation. Data was processed using Proteome Discoverer 1.4 (Thermo Scientific) and a human proteome database (UniProtUP000005640, version 2016_10, *Homo sapiens*, 70,671 entries) was searched using SEQUEST (Thermo Scientific). Search parameters included a precursor mass tolerance of 10 ppm and a fragment mass tolerance of 0.8 Da. Peptides were searched with carbamidomethyl cysteine as a static modification and oxidized methionine and deamidated glutamine and asparagine as dynamic modifications.

### Mass spectrometry data processing and bioinformatics

Two missed cleavages were permitted. The XCorr threshold value was 0.4, and the fragment ion cutoff percentage was 0.1. False discovery rate (FDR) calculations were not needed for these small datasets. There were no single peptide identifications of proteins. All original mass spectrometry data, including the number of distinct peptides assigned for each protein and the % coverage of each protein assigned, is included in Table S2. Individual replicate reports for each stable cell line were compiled into a multiconsensus report to facilitate comparisons among replicate samples. Commonly identified contaminating proteins (keratins, acetyl-CoA carboxylase alpha, acetyl-CoA carboxylase beta, pyruvate carboxylase, propionyl-CoA carboxylase, and methylcrotonyl-CoA carboxylase 1) were systematically removed. We then consulted lists of proteins identified in similar BioID studies (CRAPome (70)) and removed proteins from the reports that fit the following parameters: epitope tag BirA∗-FLAG, cell type HEK293, and affinity approach streptavidin, score of 50 or greater. Known and predicted protein–protein interactions were analyzed using STRING version 10.5 (string-db.org) and refined with literature searches. Interactions that were based on experiments rather than on text-mining or coexpression were included, and the minimum required interaction score was set to the default value (medium confidence, 0.4). MAGEL2-proximal proteins were analyzed using the Cytoscape app ClueGO to reveal functional enrichment of GO terms. The analyses included GO Biological Process, GO Molecular Function, and REACTOME Pathways.

### Immunoblotting and immunofluorescence

Immunoblots were performed as previously described (15). Primary antibodies included rabbit anti-FLAG (Sigma F7425 1:15,000), mouse anti-V5 (Abcam #ab27671, 1:5000), rabbit anti-YTHDF2 (Proteintech #24744-1AP, 1:5000), rabbit anti-USP7 (Abcam ab4080, 1:1000), and HRP-conjugated anti-actin (Sigma #A3854, 1:10,000). Secondary antibodies included HRP-linked donkey anti-rabbit IgG (Amersham Pharmacia Biotech #NA934, 1:5000) and HRP-linked sheep anti-mouse (Amersham Pharmacia Biotech #RPN4201, 1:5000). For immunofluorescence microscopy, stably transfected HEK293-MAGEL2 cells were plated onto coverslips precoated with poly-L lysine, induced in tetracycline (1 μg/ml) for 24 h, and then processed as described (15) using rabbit anti-FLAG (Sigma F7425 1:1000) or mouse anti-V5 (Abcam #ab27671, 1:1000) and Alexa Fluor 488 or 594-linked goat anti-rabbit secondary antibody (Thermofisher Scientific #A-11034 or #A-11012, 1:1000) or Alexa Fluor 488- or 594-linked goat anti-mouse (Thermofisher Scientific #A-11001 or #A-11005, 1:1000) then imaged using a Zeiss LSM 700 confocal microscope with a 40x or 63x oil immersion lens (N. A. 1.4 oil). Coimmunoprecipitation for the MAGEL2 interactors YTHDF1/2/3 was performed as previously described (134). After retaining 10% of the cell lysate as input, lysates were precleared with 20 μl Sepharose 4B beads (Sigma) for 1 h at 4 °C on a rocker. Lysates were then probed with anti-FLAG M2 affinity gel (Sigma) and mixed end-over-end at 4 °C for 2 h. Beads were washed three times (50 mM Tris-Cl pH 8.0, 150 mM NaCl, and 0.5% IGEPAL) and resuspended in 50 μl 2× Laemmli buffer (62.5 mM Tris-HCl, 3% SDS, 10% glycerol), 2% beta-mercaptoethanol, and 1% bromophenol blue. Bound proteins were eluted from beads by boiling for 10 min. Immunoblots of input samples and immunoprecipitates were performed as described above.

### Statistics for replicate stability bioassays

Continuous data are presented as mean ± SD of 3 to 4 replicates per experiment. Differences between means were evaluated using a Student’s *t* test and considered significant if *p* < 0.05. Group differences using two factors were evaluated by two-way ANOVA to determine if there was an effect of MAGEL2 expression and heat stress on the amount of YTHDF2 protein in the nucleus. Results were considered significant if *p* < 0.05.

## Data availability

Data are available on MassIVE (Mass Spectrometry Interactive Virtual Environment, ftp://massive.ucsd.edu/MSV000087525).

## Supporting information

This article contains supporting information.

## Conflicts of interest

The authors declare that they have no conflicts of interest with the contents of this article.
