# Supplementary material for: The N-terminal domain of the Schaaf–Yang syndrome protein MAGEL2 likely has a role in RNA metabolism
Source: J Biol Chem. 2021 Jul 12;297(2):100959. doi: 10.1016/j.jbc.2021.100959 (PMC8350409; doi:10.1016/j.jbc.2021.100959)
Supplement: Supplemental Tables S3–S5 [file mmc2.docx]

**Suppl. Table 3. Gene Ontology analysis of proteins in proximity to CtermMAGEL2 as detected by BioID-MS.** The 44 CtermMAGEL2-proximate proteins were analyzed using the Cytoscape app ClueGO to reveal functional enrichment of Gene Ontology terms. The analyses included GO Biological Process, GO Molecular Function, and REACTOME Pathways.

| **GOTerm** | **Group P Value** | **Associated Genes Found** |
| --- | --- | --- |
| **cadherin binding** | 1.73E-23 | AFDN, CLINT1, CORO1B, CRKL, CTTN, DDX3X, EEF2, ENO1, GCN1, GIGYF2, HNRNPK, LARP1, LDHA, MRE11, NUDC, PKM, RANGAP1, SERBP1, SLK, TLN1, UNC45A |
| **cortical actin cytoskeleton organization** | 1.37E-04 | EPB41L3, NSFL1C, TLN1 |
| platelet aggregation | 1.37E-04 | NSFL1C, POTEF, TLN1 |
| **ribonucleoprotein complex binding** | 1.25E-10 | DDX3X, DHX9, EEF2, GCN1, GEMIN5, HNRNPU, LARP1 |
| translation factor activity, RNA binding | 1.25E-10 | EEF2, EIF4A1, EIF4B, EIF4G1, GCN1 |
| ribosome binding | 1.25E-10 | EEF2, GCN1, GEMIN5 |
| **double-stranded RNA binding** | 3.23E-09 | DDX3X, DHX9, EIF4A1, EIF4B, HNRNPU |
| single-stranded RNA binding | 3.23E-09 | DDX3X, DHX9, EIF4B, HNRNPU, IFIT5 |
| RNA stabilization | 3.23E-09 | DHX9, HNRNPK, HNRNPU, LARP1, YBX1 |
| negative regulation of RNA catabolic process | 3.23E-09 | DHX9, HNRNPK, HNRNPU, LARP1, YBX1 |
| negative regulation of mRNA metabolic process | 3.23E-09 | DHX9, HNRNPK, HNRNPU, LARP1, YBX1 |
| mRNA stabilization | 3.23E-09 | DHX9, HNRNPK, HNRNPU, LARP1, YBX1 |
| negative regulation of mRNA catabolic process | 3.23E-09 | DHX9, HNRNPK, HNRNPU, LARP1, YBX1 |
| RNA helicase activity | 3.23E-09 | DDX3X, DHX9, EIF4A1, EIF4G1 |
| protein localization to cytoplasmic stress granule | 3.23E-09 | DDX3X, DHX9, YBX1 |
| CRD-mediated mRNA stabilization | 3.23E-09 | DHX9, HNRNPU, YBX1 |
| **nucleotide phosphorylation** | 1.67E-04 | ENO1, GAPDH, LDHA, PKM |
| nucleoside diphosphate phosphorylation | 1.67E-04 | ENO1, GAPDH, LDHA, PKM |
| purine nucleoside diphosphate metabolic process | 1.67E-04 | ENO1, GAPDH, LDHA, PKM |
| ribonucleoside diphosphate metabolic process | 1.67E-04 | ENO1, GAPDH, LDHA, PKM |
| ATP generation from ADP | 1.67E-04 | ENO1, GAPDH, LDHA, PKM |
| purine ribonucleoside diphosphate metabolic process | 1.67E-04 | ENO1, GAPDH, LDHA, PKM |
| glycolytic process | 1.67E-04 | ENO1, GAPDH, LDHA, PKM |
| ADP metabolic process | 1.67E-04 | ENO1, GAPDH, LDHA, PKM |
| Glycolysis | 1.67E-04 | ENO1, GAPDH, PKM |
| canonical glycolysis | 1.67E-04 | ENO1, GAPDH, PKM |
| glycolytic process through glucose-6-phosphate | 1.67E-04 | ENO1, GAPDH, PKM |
| NADH regeneration | 1.67E-04 | ENO1, GAPDH, PKM |
| NAD binding | 1.67E-04 | AHCY, GAPDH, LDHA |
| hexose catabolic process | 1.67E-04 | ENO1, GAPDH, PKM |
| glucose catabolic process | 1.67E-04 | ENO1, GAPDH, PKM |
| glucose catabolic process to pyruvate | 1.67E-04 | ENO1, GAPDH, PKM |
| glycolytic process through fructose-6-phosphate | 1.67E-04 | ENO1, GAPDH, PKM |
| **Deadenylation-dependent mRNA decay** | 1.81E-09 | EIF4A1, EIF4B, EIF4G1 |
| Deadenylation of mRNA | 1.81E-09 | EIF4A1, EIF4B, EIF4G1 |
| Translation initiation complex formation | 1.81E-09 | EIF4A1, EIF4B, EIF4G1 |
| Activation of the mRNA upon binding of the cap-binding complex and eIFs, and subsequent binding to 43S | 1.81E-09 | EIF4A1, EIF4B, EIF4G1 |
| Ribosomal scanning and start codon recognition | 1.81E-09 | EIF4A1, EIF4B, EIF4G1 |
| translation initiation factor binding | 1.81E-09 | DDX3X, EIF4G1, LARP1 |
| eukaryotic initiation factor 4E binding | 1.81E-09 | DDX3X, EIF4G1, LARP1 |
| ribosome binding | 1.81E-09 | EEF2, GCN1, GEMIN5 |
| RNA 7-methylguanosine cap binding | 1.81E-09 | EIF4G1, GEMIN5, LARP1 |
| protein localization to cytoplasmic stress granule | 1.81E-09 | DDX3X, DHX9, YBX1 |
| translation initiation factor activity | 1.81E-09 | EIF4A1, EIF4B, EIF4G1 |
| CRD-mediated mRNA stabilization | 1.81E-09 | DHX9, HNRNPU, YBX1 |
| RNA helicase activity | 1.81E-09 | DDX3X, DHX9, EIF4A1, EIF4G1 |
| regulation of translational initiation | 1.81E-09 | DDX3X, EIF4B, EIF4G1, LARP1 |
| RNA cap binding | 1.81E-09 | EIF4A1, EIF4G1, GEMIN5, IFIT5, LARP1 |
| double-stranded RNA binding | 1.81E-09 | DDX3X, DHX9, EIF4A1, EIF4B, HNRNPU |
| single-stranded RNA binding | 1.81E-09 | DDX3X, DHX9, EIF4B, HNRNPU, IFIT5 |
| translation factor activity, RNA binding | 1.81E-09 | EEF2, EIF4A1, EIF4B, EIF4G1, GCN1 |
| translation regulator activity, nucleic acid binding | 1.81E-09 | EEF2, EIF4A1, EIF4B, EIF4G1, GCN1, LARP1 |

**Supplementary Table 4. Comparison of proteins proximal to Cterminal MAGEL2, to full length MAGEL2 and Cterminal MAGEL2, or, by deduction, proximal to the N-terminal portion of MAGEL2 (MAGEL2 only).**

| Cterm  MAGEL2 only | MAGEL2 and Cterm MAGEL2 | MAGEL2 only | Role |
| --- | --- | --- | --- |
| AFDN | CLINT1 | ANKHD1 | Promote nuclear import of transcriptional co-activator YAP |
| AHCY | CTTN | ANKRD17 | Promote nuclear import of transcriptional co-activator YAP |
| CKB | DDX3X | ATXN2L | Stress granule assembly (Cluster 1) |
| CORO1B | GAPDH | CSDE1 | Stress granule assembly (Cluster 1) |
| CRKL | GIGYF2 | FUBP3 | Far Upstream Element Binding Protein (Cluster 5) |
| CTPS1 | HNRNPK | IRF2BP2 | Transcription repression |
| DHX9 | HNRNPU | KHSRP | Far Upstream Element Binding Protein (Cluster 5) |
| EEF2 | NONO | NUFIP2 | Stress granule assembly (Cluster 1) |
| EIF4A1 | NUDC | PABPC1 | Stress granule assembly (Cluster 1) |
| EIF4B | PRRC2C | PRRC2A | RNA Binding (Cluster 3) |
| EIF4G1 | SERBP1 | PUM1 | Post transcriptional repression, RNA binding |
| ENO1 | UBAP2L | SEC16A | Endoplasmic reticulum export complex (Cluster 2) |
| EPB41L3 |  | SEC24B | Endoplasmic reticulum export complex (Cluster 2) |
| EXOC4 |  | SF1 | RNA Binding (Cluster 1) |
| GCN1 |  | TNRC6A | Post-transcriptional gene silencing, RNA binding |
| GEMIN5 |  | TNRC6B | Post-transcriptional gene silencing, RNA binding |
| IFIT5 |  | UBA52 | Ubiquitination |
| LARP1 |  | UBAP2 | RNA Binding (Cluster 1) |
| LDHA |  | YTHDF1 | mRNA translation efficiency, RNA binding |
| MRE11A |  | YTHDF2 | mRNA translation efficiency, RNA binding |
| NASP |  | YTHDF3 | mRNA translation efficiency, RNA binding |
| NSFL1C |  | ZFR | Staufen shuttling, RNA Binding (Cluster 1) |
| PCM1 |  |  |  |
| PKM |  |  |  |
| POTEF |  |  |  |
| RANBP3 |  |  |  |
| RANGAP1 |  |  |  |
| SLK |  |  |  |
| TLN1 |  |  |  |
| UNC45A |  |  |  |
| USP7 |  |  |  |
| YBX1 |  |  |  |

**Suppl. Table 5. Gene Ontology analysis of proteins in proximity to MAGEL2 but not CtermMAGEL2 as detected by BioID-MS.** Functional categories are indicated.

| **GOTerm** | **Group P Value** | **Associated Genes Found** |
| --- | --- | --- |
| **regulation of translational initiation** | 7.52E-06 | DDX3X, YTHDF1, YTHDF2, YTHDF3 |
| positive regulation of translational initiation | 7.52E-06 | DDX3X, YTHDF1, YTHDF2, YTHDF3 |
| N6-methyladenosine-containing RNA binding | 7.52E-06 | YTHDF1, YTHDF2, YTHDF3 |
| negative regulation of innate immune response | 7.52E-06 | CSDE1, YTHDF2, YTHDF3 |
| **RNA stabilization** | 3.32E-05 | HNRNPK, HNRNPU, PABPC1 |
| poly-purine tract binding | 3.32E-05 | DDX3X, HNRNPU, PABPC1 |
| poly(A) binding | 3.32E-05 | DDX3X, HNRNPU, PABPC1 |
| mRNA stabilization | 3.32E-05 | HNRNPK, HNRNPU, PABPC1 |
| negative regulation of mRNA catabolic process | 3.32E-05 | HNRNPK, HNRNPU, PABPC1 |
| **regulation of mRNA catabolic process** | 6.41E-14 | GIGYF2, HNRNPK, HNRNPU, KHSRP, PABPC1, PUM1, SERBP1, TNRC6A, TNRC6B, UBA52, YTHDF2, YTHDF3 |
| regulation of RNA stability | 6.41E-14 | GIGYF2, HNRNPK, HNRNPU, KHSRP, PABPC1, PUM1, SERBP1, UBA52, YTHDF2, YTHDF3 |
| regulation of mRNA stability | 6.41E-14 | GIGYF2, HNRNPK, HNRNPU, KHSRP, PABPC1, PUM1, SERBP1, UBA52, YTHDF2, YTHDF3 |
| negative regulation of cellular amide metabolic process | 6.41E-14 | DDX3X, GAPDH, GIGYF2, KHSRP, PUM1, TNRC6A, TNRC6B, YTHDF2, YTHDF3 |
| negative regulation of translation | 6.41E-14 | DDX3X, GAPDH, GIGYF2, KHSRP, PUM1, TNRC6A, TNRC6B, YTHDF2, YTHDF3 |
| positive regulation of mRNA metabolic process | 6.41E-14 | GIGYF2, KHSRP, PABPC1, PUM1, TNRC6A, TNRC6B, YTHDF2, YTHDF3 |
| positive regulation of mRNA catabolic process | 6.41E-14 | GIGYF2, KHSRP, PABPC1, PUM1, TNRC6A, TNRC6B, YTHDF2, YTHDF3 |
| mRNA 3'-UTR binding | 6.41E-14 | HNRNPU, KHSRP, PABPC1, PUM1, SERBP1 |
| RNA destabilization | 6.41E-14 | GIGYF2, KHSRP, PUM1, YTHDF2, YTHDF3 |
| mRNA destabilization | 6.41E-14 | GIGYF2, KHSRP, PUM1, YTHDF2, YTHDF3 |
| posttranscriptional gene silencing | 6.41E-14 | GIGYF2, PUM1, TNRC6A, TNRC6B |
| **negative regulation of cellular amide metabolic process** | 6.54E-12 | DDX3X, GAPDH, GIGYF2, KHSRP, PUM1, TNRC6A, TNRC6B, YTHDF2, YTHDF3 |
| negative regulation of translation | 6.54E-12 | DDX3X, GAPDH, GIGYF2, KHSRP, PUM1, TNRC6A, TNRC6B, YTHDF2, YTHDF3 |
| positive regulation of mRNA metabolic process | 6.54E-12 | GIGYF2, KHSRP, PABPC1, PUM1, TNRC6A, TNRC6B, YTHDF2, YTHDF3 |
| positive regulation of mRNA catabolic process | 6.54E-12 | GIGYF2, KHSRP, PABPC1, PUM1, TNRC6A, TNRC6B, YTHDF2, YTHDF3 |
| posttranscriptional gene silencing | 6.54E-12 | GIGYF2, PUM1, TNRC6A, TNRC6B |
| posttranscriptional gene silencing by RNA | 6.54E-12 | PUM1, TNRC6A, TNRC6B |
| gene silencing by miRNA | 6.54E-12 | PUM1, TNRC6A, TNRC6B |
| regulation of nuclear-transcribed mRNA catabolic process, deadenylation-dependent decay | 6.54E-12 | PABPC1, TNRC6A, TNRC6B |
| regulation of nuclear-transcribed mRNA poly(A) tail shortening | 6.54E-12 | PABPC1, TNRC6A, TNRC6B |
| positive regulation of nuclear-transcribed mRNA catabolic process, deadenylation-dependent decay | 6.54E-12 | PABPC1, TNRC6A, TNRC6B |
| Oncogene Induced Senescence | 6.54E-12 | TNRC6A, TNRC6B, UBA52 |
