# Supplementary material for: The N-terminal domain of the Schaaf–Yang syndrome protein MAGEL2 likely has a role in RNA metabolism
Source: J Biol Chem. 2021 Jul 12;297(2):100959. doi: 10.1016/j.jbc.2021.100959 (PMC8350409; doi:10.1016/j.jbc.2021.100959)

Supplementary Figure 1. a) Analysis of P-X_n-G motifs in the N-terminal region of MAGEL2 compared to the intrinsically disordered protein elastin and to N-terminal region of YTHDF2. b) Schematic of the MAGEL2 N-terminus as an intrinsically disordered protein. M=MAGE homology domain, U=USP7 binding domain, red tails are the N-terminal region of MAGEL2.

Supplementary Figure 2. Validation of BirA*-CtermMAGEL2 mutant constructs. a) A 3xFLAG epitope tag and BirA* were fused in frame to the C terminal region of human MAGEL2. 3xFLAG-BirA*-CtermMAGEL2 constructs carrying p.LL1031AA and p.R1187C (black arrows) were also generated. b) Wild-type (WT) FLAG-BirA*-CtermMAGEL2 protein or mutant FLAG-BirA*-CtermMAGEL2 proteins were detected in protein lysates from stably transfected HEK293 cells induced with tetracycline by immunoblotting with anti-FLAG antibodies. The left hand-most two lanes illustrating expression of WT FLAG-BirA*-CtermMAGEL2 (compared to its mutant forms) are the same as the right hand-most two lanes of Fig. 2b that illustrate expression of FLAG-BirA*-CtermMAGEL2 (compared to full-length MAGEL2). The whole blot is shown at the bottom. c) Expression of FLAG-BirA*-CtermMAGEL2 or mutants in stably transfected HEK293 cells plated on coverslips was induced using tetracycline and visualized using anti-FLAG antibodies and confocal microscopy (green signal). Nuclei were counterstained blue with Hoechst. LL>AA is MAGEL2p.LL1031AA and R>C is MAGEL2p.R1187C. The left hand panels illustrating expression of FLAG-BirA*-CtermMAGEL2 compared to mutant forms are the same as the left hand panels of Fig. 2c that illustrate expression FLAG-BirA*-CtermMAGEL2 compared to full length MAGEL2. Scale bar 50 μm.

Supplementary Figure 3. STRING-generated interactions among proteins in proximity to CtermMAGEL2p.LL1031AA by BioID-MS. Interactions among four clusters of CtermMAGEL2p.LL1031AA proximal proteins were identified using STRING. The confidence of the predicted interaction was based on a STRING database minimum edge score of 0.4.

Supplementary Figure 4. STRING-generated interactions among proteins in proximity to CtermMAGEL2p.R1187C by BioID-MS. Interactions among seven clusters of CtermMAGEL2p.R1187C proximal proteins were identified using STRING. The confidence of the predicted interaction was based on a STRING database minimum edge score of 0.4.

Supplementary Figure 5. Comparison of MAGEL2 and CtermMAGEL2 proximal proteins. Proteins present (P) in 2 out of 3 replicates of BioID-MS with the MAGEL2 and CtermMAGEL2 protein are listed. Some of these proteins were lost (L) in all three replicates of BioID-MS with either the full length MAGEL2 or CtermMAGEL2 protein. Functional categories for proteins are also indicated.

Supplementary Figure 6. Many MAGEL2 proximal proteins are also proximal to YTHDF2. STRING diagram of proteins identified as high confidence YTHDF2 interactors by BioID, from data of Youn 2018 (100). Proteins identified by BioID as interactors of MAGEL2, CtermMAGEL2, or necdin are indicated, as is MAGED1.

Supplementary Figure 7. Co-expression of CtermMAGEL2 stabilizes YTHDF2. U2OS cells were transiently transfected with FLAG-YTHDF2 and V5-CtermMAGEL2 plasmids. *, P<0.01 by *t*-test, N=3.

Supplementary Figure 8. MAGEL2 co-immunoprecipitates with all three YTHDF proteins. U2OS cells were transiently transfected with FLAG-YTHDF1, FLAG-YTHDF2 or FLAG-YTHDF3 and either V5-MAGEL2 (M) or V5-CtermMAGEL2 (C). Protein complexes were immunoprecipitated using anti-FLAG M2 gel. 10% of the cell lysate was immunoblotted to confirm the presence of all proteins. FLAG-YTHDF1, FLAG-YTHDF2, FLAG-YTHDF3 and V5-MAGEL2 plasmids for co-transfections were constructed in pDEST-pcDNA5-FLAG or pcDNA3.1nV5-DEST using recombinational cloning from cDNAs in entry vectors.

Supplementary Figure 9. MAGEL2 and YTHDF protein expression overlaps in co-transfected cells. FLAG-YTHDF1 (a), FLAG-YTHDF2 (b) and FLAG-YTHDF3 (c) proteins were visualized (green) along with V5-MAGEL2 (red) by immunofluorescence microscopy in transiently co-transfected U2OS cells. Nuclei are stained blue with Hoechst dye. Representative cells are shown. Scale bar 5 μm.

Suppl. Fig. 1 Sanderson *et al.*

a

protein	Dist.	P-G (%)	P-X-G (%)	P-X_2-G (%)	P-X_3-G (%)	P-X_4G (%)	P-$X_{n>4}$G(%)
elastin	8.2	78.2	7.7	2.6	1.2	1.2	9.1
N-termMAGEL2 (1-819)	15.5	60	22.5	7.5	2.5	0	7.5
N-termYTHDF2 (1-403)	27.5	11.1	16.7	16.7	16.7	5.6	33.2

Dist.=average distance between P-X_n-G motifs, in amino acids, X= any amino acid

b

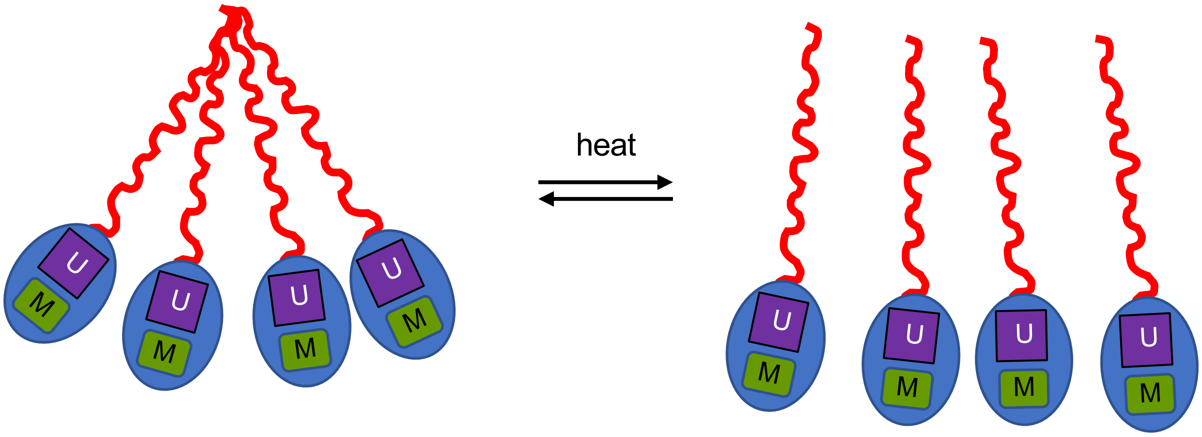

Suppl. Fig. 2 Sanderson *et al.*

a

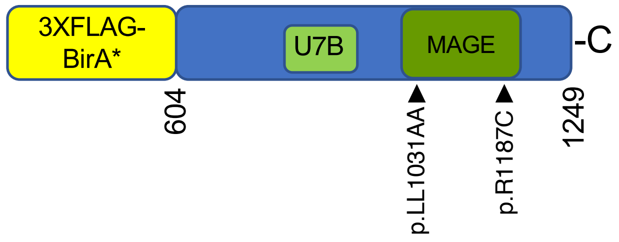

b

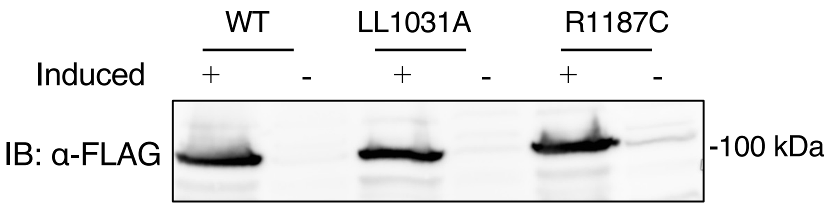

c

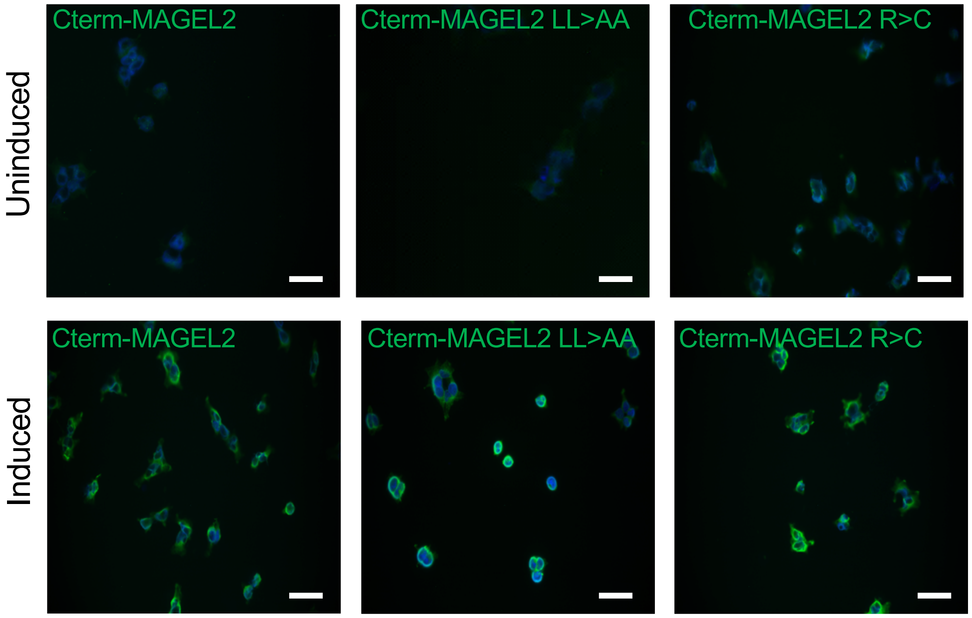

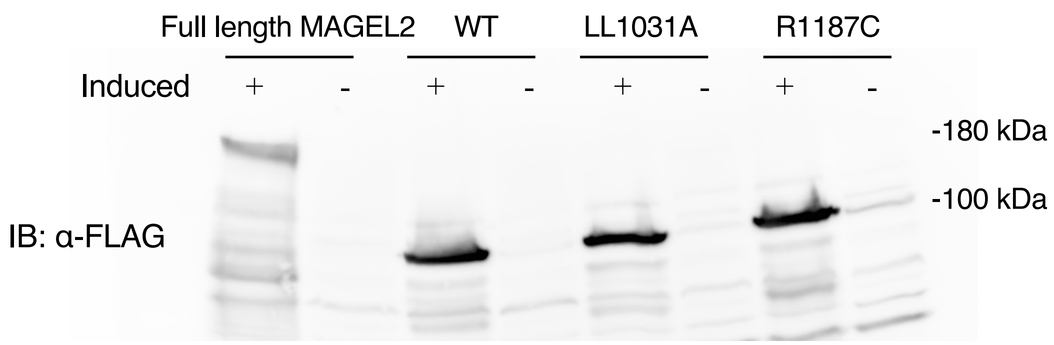

Suppl. Fig. 3. Sanderson *et al.*

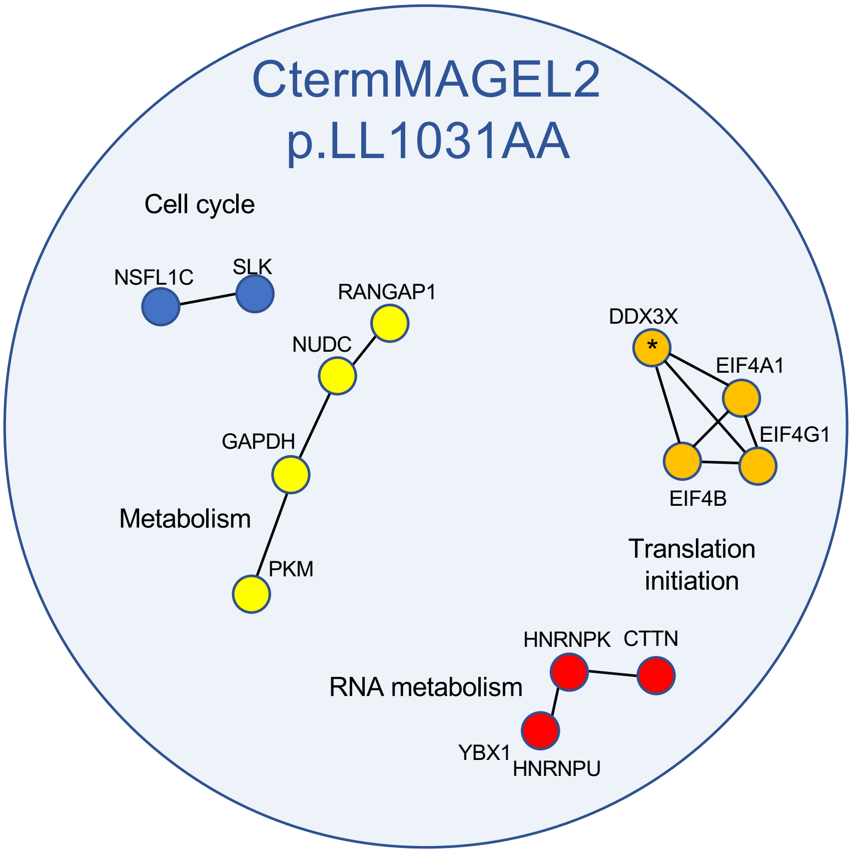

Suppl. Fig. 4 Sanderson *et al.*

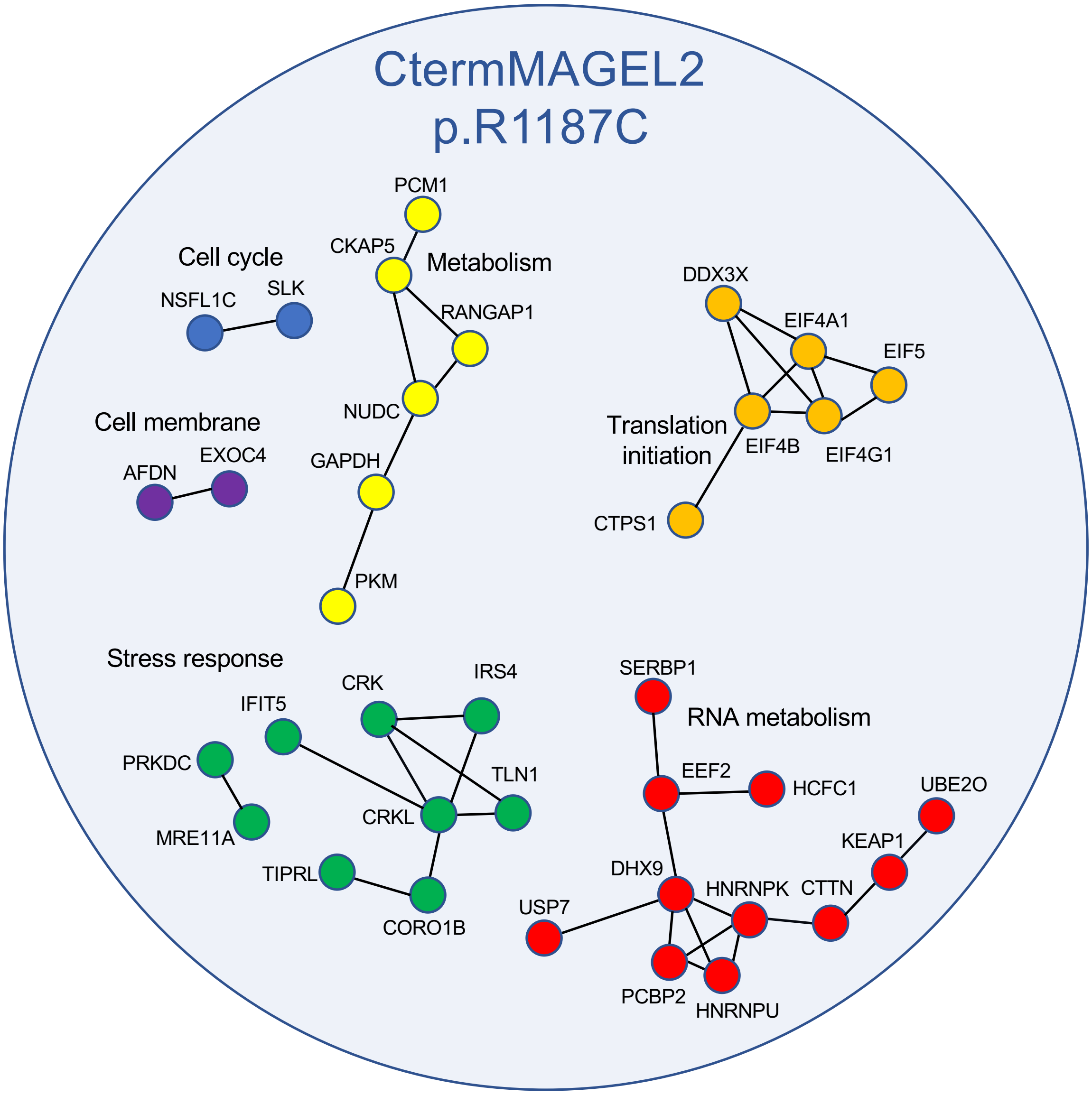

Suppl. Fig. 5 Sanderson *et al.*

	**FL MAGEL2**	**Cterm MAGEL2**	RNA metabolism	Cell signalling	Protein transport	Cell proliferation	Transcription	Translation	Cell migration	Protein degradation	Actin binding	Cell Metabolism	Protein Stability	Apoptosis	DNA Repair	Centrosome assembly	Nucelic Acid Synthesis
EIF4A1	A	P	■														
PABPC1	P	A	■														
HNRNPU	P	P	■				■										
DDX3X	P	P	■														
PRRC2C	P	P	■														
TNRC6B	P	A	■														
SERBP1	P	P	■														
LARP1	A	P	■														
YTHDF2	P	A	■														
ZFR	P	A	■														
FUBP3	P	A	■														
YBX1	A	P	■				■										
NONO	P	P	■														
NUFIP2	P	A	■														
PUM1	P	A	■														
UBAP2	P	A	■							■							
PRRC2A	P	A	■														
YTHDF3	P	A	■														
ATXN2L	P	A	■														
KHSRP	P	A	■														
HNRNPK	P	P	■														
CSDE1	P	A	■														
SF1	P	A	■														
TNRC6A	P	A	■														
YTHDF1	P	A	■														
IFIT5	A	P	■														
GEMIN5	A	P	■														
AHCY	A	P	■														
DHX9	A	P	■														
CRKL	A	P		■													
GIGYF2	P	P		■													
SEC16A	P	A			■												
SEC24B	P	A			■												
CLINT1	P	P			■												
CTTN	P	P			■												
RANGAP1	A	P			■												
EXOC4	A	P			■												
RANBP3	A	P			■												
ANKHD1	P	A				■											
NUDC	P	P				■			■								
NSFL1C	A	P				■											
NASP	A	P				■											
UNC45A	A	P				■											
ANKRD17	P	A				■											
IRF2BP2	P	A					■										
EIF4B	A	P						■									
EEF2	A	P						■									
EIF4G1	A	P						■									
GCN1	A	P						■									
CORO1B	A	P							■								
UBAP2L	P	P								■							
UBA52	P	A								■							
TLN1	A	P									■						
AFDN	A	P									■						
POTEF	A	P									■						
ENO1	A	P										■					
CKB	A	P										■					
LDHA	A	P										■					
PKM	A	P										■					
GAPDH	P	P										■					
USP7	A	P											■				
SLK	A	P												■			
EPB41L3	A	P												■			
MRE11A	A	P													■		
PCM1	A	P														■	
CTPS1	A	P															■

Suppl Fig. 6. Sanderson *et al.*

YTHDF2 BioID high confidence interactors (data from Youn et al. Mol. Cell 2018)

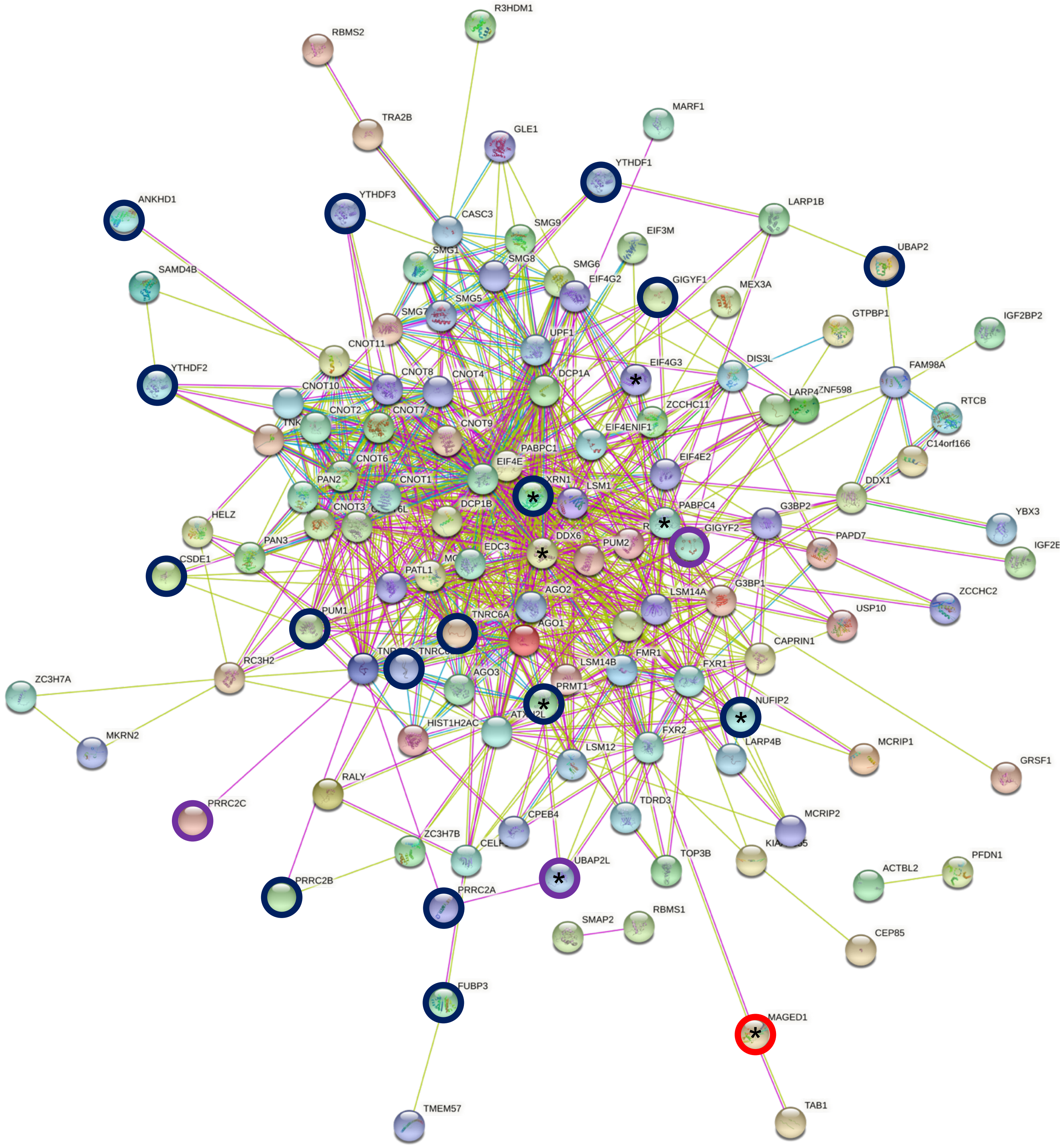

BioID MAGEL2 but not CtermMAGEL2

BioID MAGEL2 and CtermMAGEL2

MAGED1

* Necdin interactor

Suppl. Fig. 7 Sanderson *et al.*

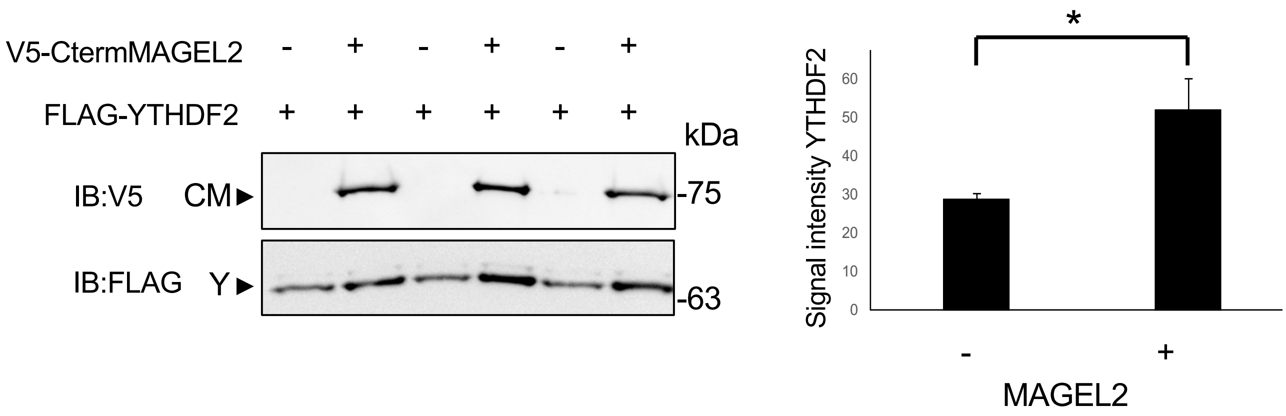

Suppl Fig. 8. Sanderson *et al.*

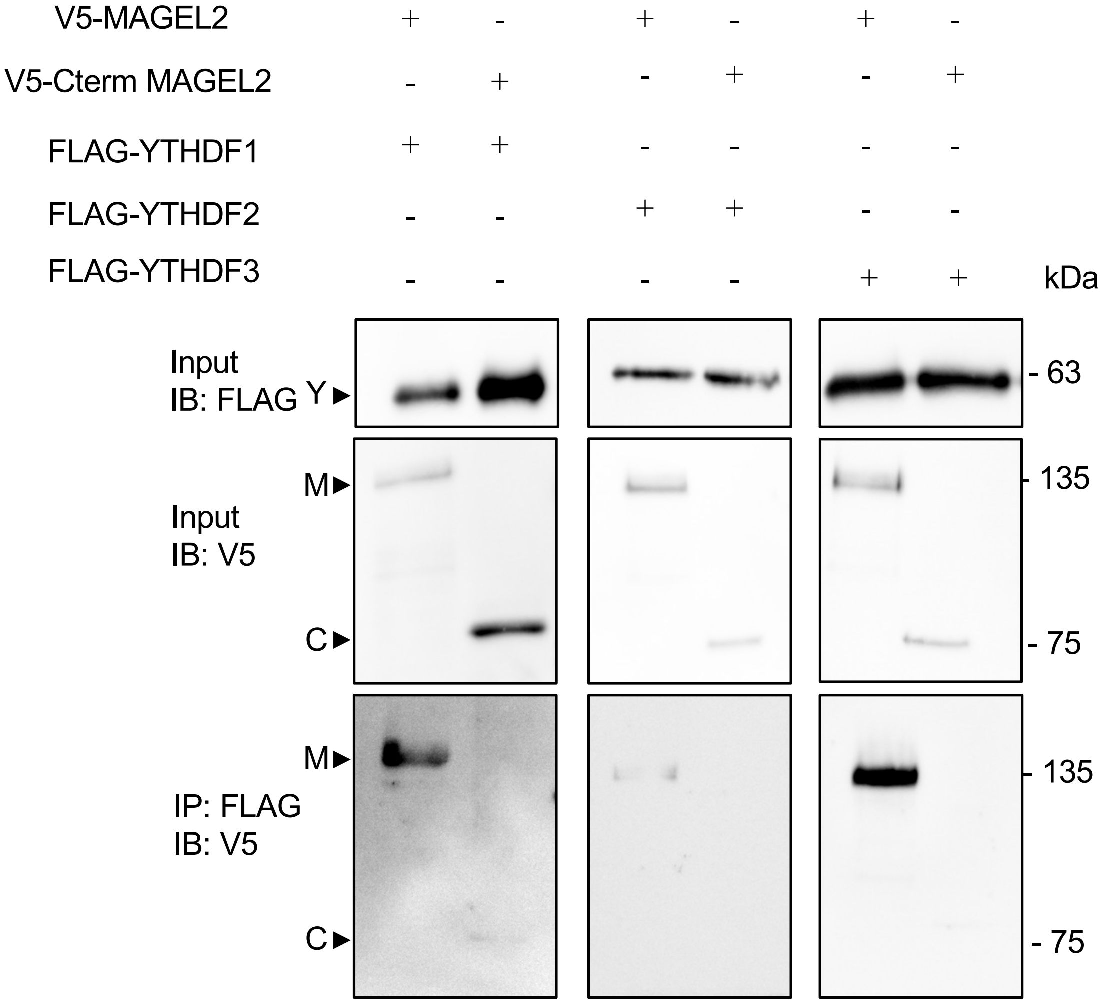

Suppl Fig. 9. Sanderson *et al.*

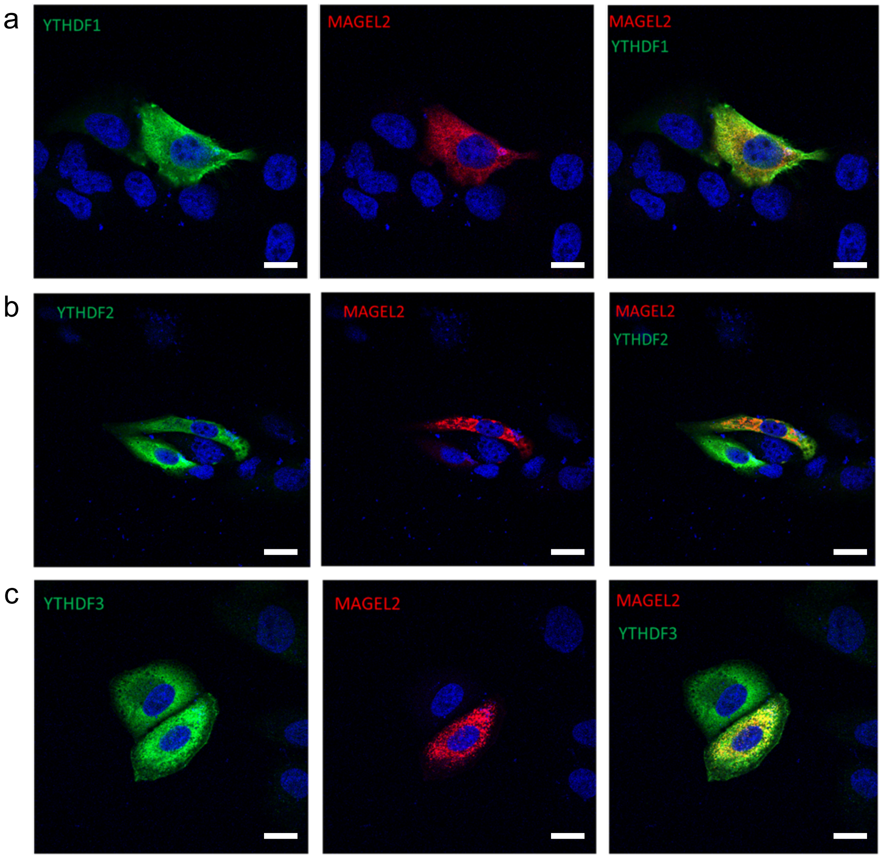

Supplement: Supplemental Figures S1–S9 [file mmc3.pdf]
